# A multi-centre, parallel-group, randomised controlled trial to assess the efficacy and safety of eurythmy therapy and tai chi in comparison with standard care in chronically ill elderly patients with increased risk of falling (ENTAiER): a trial protocol

**DOI:** 10.1186/s12877-020-1503-6

**Published:** 2020-03-17

**Authors:** G. S. Kienle, P. G. Werthmann, B. Grotejohann, K. Kaier, I. Steinbrenner, S. Voigt-Radloff, R. Huber

**Affiliations:** 1 Center for Complementary Medicine; Institute for Infection Prevention and Hospital Epidemiology, Medical Center, University of Freiburg, Faculty of Medicine, University of Freiburg, Freiburg, Germany; 2 IFAEMM at the University of Witten/Herdecke, Freiburg, Germany; 3 Clinical Trials Unit, Medical Center, University of Freiburg, Faculty of Medicine, University of Freiburg, Freiburg, Germany; 4 Institute of Medical Biometry and Statistics, Division Methods in Clinical Epidemiology, Medical Center, University of Freiburg, Faculty of Medicine, University of Freiburg, Freiburg, Germany; 5 Center for Geriatric Medicine and Gerontology Freiburg, Medical Center, University of Freiburg, Faculty of Medicine, University of Freiburg, Freiburg, Germany; 6 Institute for Evidence in Medicine (for Cochrane Germany Foundation), Medical Center, University of Freiburg, Faculty of Medicine, University of Freiburg, Freiburg, Germany

**Keywords:** Aged, Falls, Prevention, Exercise, Tai chi, Eurythmy therapy, Chronic disease

## Abstract

**Background:**

In elderly poeple, multimorbidity and polypharmacy increase while sensory, motor and cognitive functions decrease. Falls occur in 30% of people aged 65 years and older at least once per year, with injuries at 10–20%. Reducing falls and enhancing physical, emotional and cognitive capacities are essential for healthy aging despite chronic disease. Eurythmy therapy (EYT) and Tai Chi train balance, mobility and concentrative and sensory capacities.

**Methods:**

In eight trial sites (academic or community hospitals), 550 outpatients aged 65 years and older with chronic disease and increased risk of falling (history of imbalance, Berg Balance Scale (BBS) score ≤ 49) will be randomly assigned (1:1:1) to receive either EYT or Tai Chi (each provided in one-hour group sessions, twice, later once per week plus practice at home, for over 24 weeks) added to standard care or standard care alone. Standard care includes a detailed written recommendation on fall prevention and the visit of a primary care doctor. Seniors living a reclusive life or economically disadvantaged elderly will be particularly addressed. A motivation and communication concept supports the trial participants’ compliance with trial procedures and practicing. Public and patient representatives are involved in the planning and conduction of the trial. Falls will be documented daily in a diary by the participants. These falls as well as injuries and complications will be ascertained during monthly phone visits. The falls efficacy scale, BBS, cognition (MoCA), Mood (GDS-15), quality of life (SF12), instrumental activities of daily living (IADL), use of medical and non-medical services (FIMA) and adherence will be assessed at months 3, 6, and 12 and inner correspondence with practices (ICPH) at month 6. The trial is funded by the Federal Ministry of Education and Research (BMBF 01GL1805).

**Discussion:**

This study will determine whether EYT and Tai Chi reduce falls, injurious falls, fear of falling and healthcare utilisation and improve mobility, cognition, mood, quality of life and functional independence. A reduction of fall risk and fear of falling and an improvement of mobility, autonomy, quality of life, mood, and cognition are highly relevant for older people to cope with aging and diseases and to reduce healthcare costs.

**Trail registration:**

www.drks.de. DRKS00016609. Registered 30th July 2019.

## Background

Eighty percent of older adults have at least one chronic disease and 65% have at least two [1, 2], leading to an increased risk of polypharmacy [3]. Sensory functions, movement and cognitive capacity impair with aging, thus increasing immobility, frailty, dependence, health-care utilisation, risk of falling and fear of falling [3–6]. The incidence rate of falls of people aged 65–90 is 0.3–0.4 and that of ‘recurrent falls’ is 0.1 per person and year [7], and 21–85% of older adults are afraid of falling [8]. About 10–20% of falls lead to injuries, 5% to fractures and 1–2% to femoral neck fractures [5]. After femoral neck fractures, only 33–40% of patients regain their previous basic everyday competences (e.g., eating and body care), only 14–21% resume their competence for instrumental activities (e.g. shopping and phone calls) and 30% die within 1 year [5, 9]. After falls and hip fracture, 30–50% of people express their fear of falling, which reduces physical activity, mobility and self-confidence. The rates of falls, social isolation, institutionalisation and mortality have increased [5, 9]. The risk factors for falls in the elderly include the following: history of a fall, lower-extremity weakness, impairment of cognitive and sensory functions (e.g. proprioceptive sensitivity, vestibular system, vision and hearing), chronic disease (e.g. cerebrovascular or cardiovascular, Parkinson’s disease, osteoarthritis of the knee and diabetes), drugs particularly those affecting the central nervous system, type of footwear and safety hazards in the home environment [10, 11].

To interrupt the vicious circle of a physically inactive lifestyle, social isolation, reduced autonomy, increased risk of falling, morbidity [3–5, 12–14] and to enable healthy aging despite chronic disease, reducing risk and fear of falling and enhancing the physical, mental and cognitive capacities are essential [3–5]. Recommended for fall reduction and improved mobility are strength and balance training, treating osteoporosis, optimisation of medications, vision, safe homewear and environmental factors [15–17]. However, older people often do not perform the recommended exercise training, and only 14% of people aged 70–79 are physically active as recommended (at least 2.5 h per week) [18]. The reasons for avoiding exercise are health issues, negative experience and experiencing exercise like it’s a ‘chore’ and a ‘big deal’. Structured ‘artificial’ activities, such as using an exercise bicycle, usually have negative connotations. Support for regular exercise is an appreciable and encouraging social-context indoor activity that is achievable in comparison with ‘hard work’, and it begins with minimal-intensity exercises that are interesting, safe and pleasurable and that cause noticeable improvements in mood, confidence, energy and self-perceived risk of falls and injuries [19–21].

Regular Tai Chi, a mind–body intervention, can reduce falls by 19% [rate ratio (RaR): 0.81, 95% CI: 0.67–0.99], risk of falling by 20% [risk ratio (RR): 0.80, 95% CI: 0.70–0.91] and fear of falling. Altogether, exercise reduces falls by 23% (RaR: 0.77, 95% CI: 0.71–0.83) and risk of falls by 15% (RR: 0.85, 95% CI: 0.81–0.89). Although less certain, it may also reduce the number of people experiencing fall-related fractures (RR: 0.73, 95% CI: 0.56–0.95) and falls requiring medical attention (RR: 0.61, 95% CI: 0.47–0.79) [22–26]. Particularly important are exercise programmes that involve balance and functional exercises [26]. Repeating (i.e. weekly or biweekly) exercises under instruction and close observation are important for learning, training and individually tailoring the exercises to the capabilities of the elderly. Tai Chi intervention should involve at least 50 h of practice to be effective [27].

Another integrative mindfulness intervention also applied to chronically ill or elderly patients is Eurythmy therapy (EYT) [28–31]. Similar to Tai Chi, it pursues smoothly flowing multicomponent and mindfulness movements and trains balance, postural control, strength, coordination, concentration and the senses. EYT movements are linked to music, phonemes, poems or rhythms from a western cultural and emotional background. EYT is established in the outpatient and rehabilitative context and in the inpatient secondary and tertiary care and can be adapted to patients with major functional limitations. Both Tai Chi and EYT are expected to bring more awareness for slower and safer movements while performing activities of daily living. These activities are usually dual tasks (e.g. moving and carrying) and can be negatively influenced by stress or fear of falling. After the implementation of EYT as part of a training and counselling programme in construction workers, the Austrian *Allgemeine Unfallversicherungsanstalt* (General Accident Insurance Institution) found a reduction of accidents from the previous constant of 5% to zero and a reduction of sick leave [32]. In a cohort study, the disease severity and quality of life of 419 chronically ill outpatients (mostly mental and musculoskeletal conditions) improved within 3 months under EYT and were maintained for 24 months, whereas healthcare utilisation decreased [28]. A randomised controlled trial found a comparable improvement in the pain and functional capacity of chronic back pain patients under EYT, yoga and physiotherapy and a greater improvement of mental quality of life with EYT (Büssing et al. manuscript in preparation, DRKS00004651). In seven paediatric cerebellar tumour survivors, pronounced deficits in cognitive, neuromotor and visuomotor functions improved under EYT [33]. Cancer-related fatigue in breast cancer survivors improved with EYT as part of a multimodal programme [34].

Integrative and mind-body-based interventions have gained increasing importance and popularity. Particularly, people entering the age of 60+ can be expected to have a strong preference for integrative mind-body interventions compared with the artificial training of strength and balance [35–37]. As actual training depends on motivation and availability, an assessment of these interventions in health outcomes and inner involvement in chronically ill elderly people with increased risk of falling is of major importance. The underlying question is whether mindfulness exercise can interrupt the vicious circle of chronic disease, falls, inactivity, loss of independence and reduced quality of life.

### Objectives

The primary objective is to determine whether EYT and Tai Chi can reduce the risk of falling (i.e. experiencing at least one fall), which usually marks the beginning of dependency and the decline of mobility. This is assessed by comparing EYT plus standard care, Tai Chi plus standard care and standard care alone with respect to the risk of falling over a time period of 6 months. The secondary objective is to determine whether EYT and Tai Chi can improve mobility and balance and reduce injuries through falls and fear of falling, which are usually the primary threat to mobility. The follow-up question is to determine whether this leads to the better management of daily tasks and a better health-related quality of life, cognition and mood and whether it affects healthcare utilisation and costs. Finally, the inner correspondence of the participants with the practices and how well they adhere to them will be assessed.

### Study design

Multi-centre, randomised controlled, open-label, confirmative trial with two active and one control parallel groups.

## Methods: participants, interventions and outcomes

### Study setting and time schedule

Eight qualified trial sites are used in this trial: Medical Center – University of Freiburg (Centre for Complementary Medicine), Tübingen, (Institute of General Practice and Interprofessional Care), Witten Herdecke (Institute of Integrative Medicine), Duisburg-Essen (Department of Naturopathy and Integrative Medicine), Ulm (Integrative Medicine), Charité, Berlin (Immanuel Hospital), Havelhöhe Hospital, Berlin and Filderklinik, Filderstadt. The University Centre for Complementary Medicine and the Clinical Trial Unit (CTU) of the Medical Centre–University of Freiburg organise and manage the trial. Patient recruitment is planned from the third quartile of 2019 to the second quartile of 2021. The follow-up will end in the second quartile of 2022.

### Key eligibility criteria

Included will be adults who are at least 65 years, have a chronic disease (e.g. musculoskeletal, neurological or cardiovascular), have an increased risk of falling [Berg Balance Scale (BBS) score 49 or less and a self-reported history of imbalance], are able to participate in 1 h group-based sessions and are able to leave home on their own. There is no restriction with respect to their domicile.

Excluded will be patients with medical conditions limiting participation (e.g., terminal illness, heart failure NYHA III-IV, unstable angina pectoris, uncontrolled seizure disorder, decompensated lung disease, cancer in the advanced stage, chemo- or radiotherapy ongoing or during the last 3 months or amputation of one or both legs); complete dependence on a rollator (wheeled walker); major visual, hearing or language problems affecting the understanding of trial documents and procedures; major cognitive impairment (MoCA score 18 or less); conditions affecting the participation of group classes (e.g. high risk for individual support, urge incontinence, personality disorder and substance use disorders); life expectancy less than 1 year; permanent confinement to bed to be expected in less than a year; participation in regular Tai Chi or EYT exercises within the last 6 months and participation in vigorous sports for exercise within the last month.

A specific recruiting strategy will strengthen the enrolment of elderly people who are economically disadvantaged and living a reclusive life.

### Interventions

Participants will be randomly allocated to receive standard care plus EYT or plus Tai Chi or standard care only (control).^1^

#### What

EYT [30, 31] and Tai Chi [22–25] exercises are described in a manual. They are derived from previous studies investigating fall prevention or based on recommendations by experts. They have been reviewed by Tai Chi teachers, EYT therapists and external experts.

‘Standard Care’: participants receive a well-described brochure that recommends evidence-based measures to reduce risk of falling (e.g. training balance, strength, flexibility, endurance; medication modification; safe home environment; bone health; safe footwear; assistive devices) and provides addresses for more information or support [38]. Participants will also be recommended to visit their primary care physician to receive care based on evidence-based guidelines to prevent falls [39] or to manage multi-morbidity [40] or polypharmacy [41].

#### Who provides

EYT is instructed and guided by a certified EYT therapist (5 1/2 years of training according a standardised curriculum). Tai Chi is instructed and guided by a teacher with at least 500 h of training according to a standardised curriculum or an equivalent of 500 h of own teaching. Therapists and teachers are experienced in instructing and guiding the exercises. They are invited to practice together before the commencement of the trial. Standard care is provided and organised by the participants’ primary care doctor or consultant according to the patients’ own discretion. The brochure [38], which also enables participants to conduct self-help, is handed over by the trial sites.

#### How

EYT and Tai Chi will be provided in one-hour sessions, in groups of 4–6 participants, and may include a resting time (approximately 15 min) in the end. For practicing at home, participants receive oral, written (manual) and audio-visual instructions.

#### Where

Group classes will be held in appropriate rooms of sufficient size, which provide one stable and safe chair per participant and are accessible for those with disabilities, within reach of public transportation and, if possible, located nearby the participants. For home training, participants receive safety instructions.

#### When

Group sessions will take place twice a week during weeks 1–12 and once per week during weeks 13–24. The intensity is higher in the first weeks, taking into account the learning curve, and is reduced later when individual practicing has become routine. The participants are instructed to practice exercises at home for a minimum of 20 min during three non-class days.

#### Tailoring

Tai Chi and EYT follow the manuals but can be individually tailored to the capabilities of the participants.

#### How well

The participants will keep training diaries. The therapists and teachers will keep attendance lists and lists of deviations from the planned exercises.

#### Additional interventions

All other treatments are allowed outside the trial except EYT or Tai Chi/Qigong/Yoga in the non-EYT or non-Tai Chi groups, respectively.

### Outcomes

*Primary outcome* Risk of falling is the incidence of experiencing at least one fall within 6 months. A fall is defined as ‘an unexpected event in which the participants come to rest on the ground, floor or lower level’ [42]. The frequency of falls is self-reported daily in the fall diaries and ascertained during the monthly phone calls. Its assessments follow international guidelines [42].

*Secondary outcomes* are the risk of falling at month 12; the number of falls and injurious falls categorised into ‘moderate’ (sprains, bruises, scrapes, joint injuries and needing medical care) and ‘serious’ (fracture, head injury, tendon rupture, inpatient care and requiring stitches) [43]; balance and mobility (BBS [44]); cognition (MoCA [45]); fear of falling (short FES-I [46]); mood (GDS-15 [47]); health status (SF-12 [48]); instrumental activities of daily living (IADL [49]); use of medical and non-medical services (FIMA [50]); medications (total number and classes of fall-risk-increasing drugs [51]); adherence to practicing in groups and at home (participants’ lists and training diary); inner correspondence/peaceful harmony with practices (ICPH [52]), use of standard fall prevention measures and subjective estimation of motivational support; and contamination with Tai Chi or EYT in the non-Tai Chi and non-EYT groups (self-designed questionnaires, see Supplementary file 1, 2, 3 and 4)).

### Complications

The complications that can arise from EYT, Tai Chi or other exercises will be collected monthly. A complication is any untoward medical occurrence such as any unfavourable and unintended sign, symptom, or disease that is possibly related to the study intervention. Not regarded as complications are conditions that were already present at the time of the informed consent or aching muscle, which is to be expected. Starting with the open questions, participants are asked about any untoward medical occurrences or unplanned medical visits. In case of a possible relation to the intervention, these occurrences are documented, including severity and outcome. Moreover, therapists or teachers can inform the trial sites about the complications. Falls and injurious falls are documented as primary outcomes, and injuries are reported as complications in case they occurred during training. Further information on safety will be derived from the FIMA, which assesses healthcare utilisation such as physician contacts and hospitalisations.

### Participant timeline

The time schedule of enrolment, interventions, assessments and visits for participants is shown in Table 1.

**Table 1:** Participant timeline of the visits and assessments

VISITS	Pre-Treatment (Screening, Waiting time, Baseline/Visit 1 Randomisation)				Treatment					Post-Treatment Follow-up	
						Phone Visit 2–3	Visit 4	Phone Visit 5–6	Visit 7	Phone Visit 8–12	Last Visit, EOT
**TIME**			**Week − 4 to Day − 1**		**Day 0**	**Month 1 & 2**	**Month 3**	**Month 4 & 5**	**Month 6**	**Month 7–11**	**Month 12**
**Pre-Screening**	**X**										
**Waiting time**		**X**									
**Randomisation**				**X**							
**Informed consent**			**X**								
**In-, exclusion criteria**			**X**								
**Demographic Data**			**X**								
**Handing out Study Book**					**X**						
**Fall prevention information**					**X**						
**Recommendation to visit primary care doctor**					**X**						
**Start treatment or control per participant**					**X**						
**Review fall diary**						**X**	**X**	**X**	**X**	**X**	**X**
**BBS**			**X**				**X**		**X**		**X**
**MoCA**			**X**				**X**		**X**		**X**
**IADL**			**X**				**X**		**X**		**X**
**Medications**			**X**				**X**		**X**		**X**
**Baseline Questions**			**X**								
**Short FES-I**			**X**				**X**		**X**		**X**
**GDS-15**			**X**				**X**		**X**		**X**
**SF-12**			**X**				**X**		**X**		**X**
**ICPH (only EYT and Tai Chi group)**							**X**		**X**		
**FIMA**			**X**				**X**		**X**		**X**
**Safety; complications**						**X**	**X**	**X**	**X**	**X**	**X**
**Adherence, motivation, additional interventions**									**X**		

### Sample size

Sample size calculation is based on the assumptions on the proportion of participants with at least one fall during a six-month period. The proportion of participants with at least one fall is assumed as 50% in the standard care group [44, 53]. A decrease of this proportion to 35% is considered clinically relevant and achievable by EYT and Tai Chi [54]. The study is planned with 80% power to show the difference between treatments at the two-sided level alpha of 5% when the proportions of fallers (i.e. participants experiencing at least one fall within 6 months) is 50% with standard care and 35% with both EYT and Tai Chi. According to the chi-square distribution, 155 participants per group (calculated with NQuery Advisor 7.0) are required. The sample size calculation is performed for the three-group comparison. To account for multiplicity issues in this three-group comparison, a closed test procedure at the two-sided level alpha of 5% will be used. That is, the global null hypothesis of no differences between any of the three intervention types will be tested first, and if it can be rejected, pairwise group comparisons will be conducted each at the two-sided level alpha of 5%. The two-group comparison of EYT versus standard care has a power of 80% when the proportion of fallers is reduced from 50 to 34.3%. The two-group comparison of EYT versus Tai Chi has a power of 80% when the difference in the proportion of fallers is 15%. To account for a certain amount of non-compliance and incomplete observations, 550 participants will be randomised at a ratio of 1:1:1.

### Recruitment

The trial will be announced through local media, a web page (https://www.uniklinik-freiburg.de/entaier-studie.html), flyers, posters and personally to elderly people and to key people in contact with the elderly (senior citizens, physicians, hospitals, pharmacies, ambulances, nursing services, household help, food banks, social workers, social welfare office).

Potential participants contacting the local trial site will be pre-screened through a phone call. Elderly people that appear to be eligible will enter a waiting time until 15 or 30 participants could be enrolled. Only then will they receive detailed information. After providing their written consent, they are screened, enrolled and assessed for the baseline by the local trial sites. Within four subsequent weeks, they are randomly allocated to the three groups and receive their study book containing the dairies and all relevant information. This step initiates the observation period and is immediately followed by the start of the EYT and Tai Chi sessions.

## Methods: assignment of interventions

### Randomisation

Central randomisation by fax will be performed in this trial (CTU). Randomisation will be stratified by clinical site and performed in blocks of length 12, 15 or 18 at a ratio of 1:1:1. The randomisation code will be produced by validated programmes based on the Statistical Analysis System. For each batch size (12, 15 and 18), a separate randomisation fax form and randomisation lists will be prepared.

### Blinding and minimising risk of bias

The blinding of the Tai Chi and EYT group and the standard care group to their allocation is not possible because it is obvious in which group the participants are randomised, as all three trial interventions differ substantially. The outcomes are either directly reported by the participants (falls, questionnaires) or in close communication with the participants (falls, complications, BBS, MoCA, IADL). Therefore, also a reliable blinding of the outcome assessment to participant allocation is not possible. Outcome assessors are instructed by a written guide to start with open and general questions and to avoid mentioning the group allocation as long as possible. Since the electronic case report forms differ depending on randomized treatment, the statistical analysis will not be blinded, but all confirmatory analyses are prespecified in the clinical trial protocol and decisions on analysis data sets will be finalized without access to the unblinded data. Concealment of the randomisation will be guaranteed to minimise selection bias. All assessments will be conducted in a comparable setting and time. The primary outcome parameter is clearly defined and described in detail [42]. The combination of prospective, daily, participant-completed falls diary and monthly calls is regarded to be reliable and captures most of the falls among seniors [55]. To reduce incomplete outcome data, travel costs for public transportation are refunded, and the trial sites may conduct the follow-up visits at the participants’ home. Two of the groups receive interventions in a comparable setting, support and frequency. The third (control) group, although not offered regular training sessions, can still avail of such opportunities through the addresses in the brochure, the general courses in their city or their primary care physician. They receive human attention through an appreciating communication with the trained study personnel and their primary care doctors, other consultants and medical referrals at their discretion and informed by evidence. The patient informed consent (form) makes no remarks on the potential efficacy of EYT or Tai Chi to reduce expectancy bias. Publication bias will be reduced by study registration in the trial registers, the publication of the trial protocol and the results and the support of open data.

## Methods: data collection, management and analysis

### Data collection methods

BBS, MoCA, IADL and the complications are assessed by the study personnel.

The FES-I, GDS-15, SF-12, ICPH and FIMA questionnaires, the falls and training diaries and the self-designed questionnaires (see Supplementary file 1, 2, 3 and 4) are filled in by the participants in the same environment, which is quiet and respectful of the subject’s privacy. As much as possible, the outcome measures are assessed at a consistent time of the day and consistent with the medication cycles, particularly in participants with conditions like Parkinson’s and multiple sclerosis. The data obtained by the investigators are kept in source documents and directly entered in a trial-specific electronic case report form (eCRF). The patient-reported questionnaires are stored as the participants’ source documents, and a duplicate is sent to the CTU in Freiburg to be entered in the eCRF.

### Retention

Participants will be compensated for their travel costs: €110 for the first 6 months and €20 each for the screening and follow-up. If necessary (e.g. social welfare)*,* up to 15% of participants can receive further reimbursement of public transportation costs to visit group classes. Compliance with the trial procedures will be supported through appreciation, expressed gratefulness, giving participants sufficient time to be heard and seen, detailed explanation of the purpose and necessity of such studies to improve elderly care and a small token during every visit of the trial site (e.g. a post card with motifs from art, nature or aphorisms).

Participants may withdraw from the group classes or from the study for any reason at any time. The investigator may withdraw a participant from the intervention if the risks outweigh the benefit or if the inclusion/exclusion criteria are severely violated. Nevertheless, further follow-up visits and the assessment of the trial endpoints will be attempted to conduct an analysis according to the intention-to-treat principle.

Following the recommendation of public and patient representatives (PPR), EYT therapists, Tai Chi teachers and study personnel will receive a 3 h personal and written refresher training in appreciating, encouraging, non-judgmental communication and managing challenges of the group situation provided by a communication trainer. The EYT therapists and Tai Chi teachers will also receive a motivation concept on elderly people to comply with regular exercises, with a specific focus on the elderly living a reclusive life. The motivation concept is based on published research [21, 56, 57–61] and expert consultation.

### Data management, quality control and monitoring

Extensive procedures ensure the quality control of data collection, data entry and subject confidentiality. The study data are collected and managed using REDCap™ Version 8.6.5, a fully web-based remote data entry system based on web forms developed and maintained by the REDCap Consortium (redcap@vanderbilt.edu).

Data entry and data corrections on the e-forms are automatically tracked in an audit trail created by the electronic data capture system. Data will be regularly reviewed for completeness, consistency and plausibility. During the trial, the clinical research associate will visit the trial sites regularly. The clinical research associate will verify if the trial is conducted according to the trial protocol (signed informed consents, eligibility of participants, primary endpoint and safety data), the trial-specific procedures, the International Conference on Harmonisation-Good Clinical Practice guideline (ICH-GCP, R2) and the national/local regulatory requirements and will compare the entries in the eCRF with the source data.

Data will be exported from the database into the statistical software for analysis.

### Statistical analysis

The primary efficacy analysis will be performed according to the intention-to-treat principle. All randomised participants will be analysed as belonging to their randomised arm, regardless of whether they refused therapy or whether other protocol deviations are known.

For the analysis of the primary endpoint fall (yes, no) within 6 months, a time-to-event methodology will be applied using the time to the first fall to account for the potentially incomplete observation times of the participants. This is considered to be more efficient than an analysis of the binary endpoint fall (yes, no), therefore leading to a gain in power. For participants who do not fall within the six-month observation period, the time to last contact will be used as censored observation. Death without a previous fall will be regarded as a competing event in the analysis. A Cox regression model will be used to test the efficacy of EYT versus Tai Chi versus standard care in reducing the risk of falling within 6 months after handing out the study book. The model will include the intervention type, trial site, BBS score and age as the independent variables. To account for multiplicity issues in this three-group comparison, a closed test procedure at the two-sided level alpha of 5% will be used. That is, the global null hypothesis of no differences between any of the three intervention types will be tested first, and if it can be rejected, pairwise group comparisons will be conducted each at the two-sided level alpha of 5%. Hazard ratios between the interventions will be calculated from the model with a two-sided 95% CI. In addition to the overall comparison of the treatment arms, a comparison of treatments in the key participant subgroups will be of interest. The treatment effect will be estimated separately in the participants defined by different diagnoses (present vs. absent) from the regression models, including the interaction between treatment and diagnosis.

For the analysis of the secondary endpoint total number of falls per participant within 6 months after handing out the study book, a negative binomial regression model will be used to test the efficacy of EYT versus Tai Chi versus standard care. A negative binomial is preferred over a Poisson regression because of the potential overdispersion. Fall rates for participants with follow-up times of less than 6 months will be adjusted by accounting for their exposure time. The model will include intervention type, trial site, BBS score and age as the independent variables. The incidence rate ratios between interventions will be calculated from the model with a two-sided 95% CI. To consider the potentially present excess of zeroes, a zero-inflated negative binomial model will be used as a sensitivity analysis. Other secondary endpoints will be analysed with the appropriate regression models.

Safety analyses will be performed for all participants for whom one of the interventions is started, and participants will be analysed according to the intervention received. The rates of adverse events and serious adverse events will be calculated with a two-sided 95% CI. No formal interim analyses of the efficacy endpoints will be performed.

### Economic analysis

The utilisation of healthcare cost will be calculated by applying a standardised unit cost allowing the evaluation of cost development for each group over time. The costs of healthcare utilisation (months 6 and 12) will be analysed using appropriate regression models. Intervention type, trial site and utilisation costs at baseline will be included as the independent variables.

In the second step, cost-effectiveness analyses of the intervention will be conducted from the healthcare perspective. Therefore, the development of the different endpoints will be analysed in relation to the costs of healthcare utilisation and the costs of the intervention. Incremental cost effectiveness ratios and the corresponding confidence intervals will be estimated.

## Methods: independent committees

### Data monitoring committee (DMC)

A DMC comprises four specialists with high expertise in conducting clinical trials in Tai Chi and EYT and in safety assessment and falls in the elderly. These specialists are independent from the study organisers and all trial sites. They will regularly review the complications of the trial treatments and advise the principal investigator (the details are described in the DMC charter).

### Public and patient involvement

The PPR are involved during the planning, conduction and analysis of the trial following the recommendations by INVOLVE (http://www.invo.org.uk). Through this, the views, values, needs, strengths and limitations of the older people are integrated. In the personal semi-standard interviews, the details of the trial design are explained and discussed. Their comments and recommendations centre on motivation; how to recruit older people, elderly leading a reclusive life and people from a lower socioeconomic background; and the importance of an appreciating, encouraging and calm atmosphere, the giving of sufficient time and avoiding the sense of being in need of help. Audio-visual material to support practicing at home is also recommended. Therefore, communication training, the provision of audio-visual material and the details of the recruitment strategy are added, and more time is planned for the visits. The PPR are invited to all relevant trial meetings and receive relevant trial documents, analyses and trial reports. Particularly, the documents handed out to participants (i.e. brochure, audio-visual material, information sheets, flyer and web page information) and their adequacy, comprehensibility and readability are discussed in detail. The PPR will be informed about the progress of the trials and unexpected problems and will be consulted regarding participant recruitment.

## Methods: ethics and dissemination

### Ethical and legal principles

Before enrolment, the potential participants will be informed about the goals, procedures and possible risks of the trial; data protection and confidentiality; and the voluntary nature of the participation throughout the study. A written informed consent must be obtained for all potential participants before they are registered. Before the study begins, approval from the local ethic committees will be obtained. (From the Ethics Committee of the University of Freiburg, the ethical approval has been obtained: 183/19; 23.07.2019, as well as from the Ethics Committees of Tübingen for the sites Tübingen and Filderstadt, 561/2019BO1, 21.10.2019, Witten/Herdecke 150/2019, 31.07.2019 and Ulm 284/19, 01.08.2019; Berlin relies on the vote from Freiburg). The study will be carried out in compliance with the Declaration of Helsinki and the ICH-GCP.

### Confidentiality

Data protection follows the data protection concept of the CTU and must be fully and permanently complied with. The names of participants are only available in the source documents, which are kept in a secure and lock-protected location at the trial sites and in the participation lists of the EYT therapists and Tai Chi teachers. The findings obtained in the course of the study will be stored in electronic media and treated in strict confidence. For the protection of these data, organisational measures are taken to prevent disclosure to unauthorised third parties. For example, the subject data will be captured in pseudonymised form (i.e. subject ID number for the particular study and year of birth) throughout the documentation and evaluation phases.

### Ancillary care

The participants enrolled in the trial are included in an insurance that covers the expenses in case a participant suffers harm from trial participation.

### Access to data and dissemination policy

The principle investigator has full access to the final trial dataset. The trial results, treatment manuals and videos will be published and presented to healthcare professionals, the public and other relevant interest groups. The full trial protocol is accessible by the public (see Supplementary file 5). The principle of open data is supported (https://www.bihealth.org/de/quest-center/mission-ansaetze/open-science). Anonymised data may be shared with cooperating scientists or scientists who have other medically or scientifically well-founded reasons (goals referring to transparency, replicability, different analyses, combinations and meta-analyses of data). Their research must aim at improving the care of elderly people, and the researchers have to ensure data protection.​.

## Discussion

Falls are a major threat for elderly people, and they usually mark the end of their independence [6]. About 80% of women aged 75 or older prefer death to a ‘bad’ hip fracture, which can result in loss of independence and nursing home admission [62]. Falls, even if non-injurious, lead to a decline in social activities, mobility, and cognitive and physical performance [63, 64]. In European countries, North America and Australia, 0.85–1.5% of the total national healthcare expenditures are spent on fall-related costs, which correspond to 0.07–0.20% of the gross domestic product [65]. Therefore, reducing the risk of falls and fear of falling and increasing mobility, physical fitness and activity can be a significant benefit for elderly people, providing them with functional independence, autonomy and better life quality and reducing morbidity, costs and healthcare utilisation.

This work is the first trial investigating EYT in elderly people with high risk of falling. We expect the effects of EYT on falls to be similar to the effects of Tai Chi, but to be different with regard to preferences, memorability of exercises, adherence, mood and quality of life. The patient’s perspective, treatment context and adaptability to physical fitness are important for motivation [19–21]. Although Tai Chi has been frequently investigated internationally, this trial is the first trial to examine Tai Chi in Germany in this population and indication.

The strengths of the ENTAiER trial lie in the highly relevant issues of aging population, increasing burden of mostly lifestyle-related multimorbidity and high risk of falls increasing morbidity, mortality and costs; the methodological rigor, the multi-disciplinary team and its high competence and experience; and the data quality and conformity to the ICH-GCP. Moreover, the active and constant involvement of the PPR improved the trial quality and its consequent adjustment to patients’ needs, values, goals and capabilities. Transparency is highly valued in this study. Aside from the PPR, the design has been discussed with many experts in the field, organisations representing or supporting senior citizens and healthcare providers. The treatment manuals and videos will be published after the termination of the trial to enable replicability or to implement them in future care. They further enable the autonomy of participants for independent practicing beyond group classes. We attempt to enrol older people who lead a reclusive life, are economically disadvantaged and have an increased risk of falling [51] but seldom visit exercise classes or other activities of their own accord. The participation of these people is supported by a specific motivational concept and by an appreciating and non-judgmental communication through the study personnel, teachers and therapists.

The ENTAiER trial has some limitations. First, the focus of the trial tends towards a pragmatic assessment at the expense of explanatory interpretation. Therefore, the result will assess the combination of exercises and teachers’/therapists’ ability to instruct, motivate and individually tailor exercises. The differences between therapists cannot be captured. However, more than 20 therapists and 20 teachers in different settings (trial sites, places for group settings and groups) will provide EYT and Tai Chi, levelling out the effects of specific personalities and settings. We will also monitor the adherence to home and group training, the adherence to and adaption of exercises by the therapists and teachers, the participants’ subjective impression of what motivated them and the inner coherence with practices.

No blinding is possible for exercise classes, and no intervention mimicking EYT or Tai Chi while lacking any specific effects for fall prevention can be found. Potential competing interventions, such as stretching, life reviewing groups and training in health competencies and literacy, if well designed and conducted, all have specific effects on the risk factors related to falls. However, if they are clearly designed to lack all specific effects on any risk factors, then they are ethically questionable, and the PPR voted against them. A waiting list control has been discussed to have nocebo effects, leading to an overestimation of the effects [66]. Therefore, a three-arm design, which compares a highly investigated versus a scarcely investigated intervention versus a standard care only group, is chosen.

We may document more complications in the EYT and Tai Chi groups in comparison with the control group in case the participants in the intervention groups practice more often and more intensely. Therefore, more untoward medical events may be potentially linked to the exercises. We reduce this bias by presenting open questions and avoiding any influence through suggestive questions. Furthermore, we would be more concerned by an underreporting of potential complications, which is not to be expected. The net risk necessitating the medical and non-medical services (FIMA) will be regularly assessed without risk of bias.

## Supplementary information

**Additional file 1: Supplementary file 1**. Self designed questionnaire Initial English_ENTAiER: Initial Questions.

**Additional file 2: Supplementary file 2**. Self designed questionnaire Initial German_ENTAiER: Eingangsfragen.

**Additional file 3: Supplementary file 3**. Self designed questionnaire month 6 English_ENTAiER: Questions About the Last 6 Months.

**Additional file 4: Supplementary file 4**. Self designed questionnaire month 6 German_ENTAiER: Fragen zu den letzten 6 Monaten.

**Additional file 5: Supplementary file 5**. CTP_V1.1_ENTAiER_20190601: A multi-centre, parallel-group, randomised controlled trial to assess the efficacy and safety of Eurythmy Therapy and Tai Chi in comparison to standard care in chronically ill elderly patients with increased risk of falling (ENTAiER), Version 1.1, 2019-06-01.

## Data Availability

The principle of open data is supported (https://www.bihealth.org/de/quest-center/mission-ansaetze/open-science). Anonymised data may be shared with cooperating scientists or scientists who have other medically or scientifically well-founded reasons (goals referring to transparency, replicability, different analyses, combinations and meta-analyses of data). Their research must aim at improving care of elderly people, and they have to ensure data protection.

## Abbreviations

BBS: Berg Balance Scale
CI: Confidence Interval
CONSORT: Consolidated Standards of Reporting Trials
CTU: Clinical Trials Unit
DMC: Data Safety and Monitoring Board
eCRF: electronic Case Report Form
ENTAiER: Elderly Need Tai Chi and Eurythmy
EYT: Eurythmy Therapy
FAS: Full Analysis Set
FIMA: *Fragebogen zur Inanspruchnahme Medizinischer und Nicht-medizinischer Versorgungsleistungen im Alter (Questionnaire for Health-Related Resource Use in an Elderly Population)*
FRIDS: Fall Risk Increasing Drugs
GDS-15: Geriatric Depression Scale
h: hours
IADL: Self-Maintaining and Instrumental Activities of Daily Living
ICH-GCP: International Conference on Harmonisation-Good Clinical Practice
ICPH: Inner Correspondence/Peaceful Harmony with Practices
MoCA: Montreal Cognitive Assessment
NYHA: New York Heart Association
PPR: Public and patient representatives
RaR: Rate ratio
RR: Risk ratio
SF-12: Short Form Health Survey, 12 items
Short FES-I: Short Falls Efficacy Scale-International
TIDieR: Template for intervention description and replication

## Footnotes

1 For practical purposes, the three groups will be referred to in this trial protocol as EYT, Tai Chi and Standard Care only, although standard care is present in all three groups.
