# Supplementary material for: A multi-centre, parallel-group, randomised controlled trial to assess the efficacy and safety of eurythmy therapy and tai chi in comparison with standard care in chronically ill elderly patients with increased risk of falling (ENTAiER): a trial protocol
Source: BMC Geriatr. 2020 Mar 17;20:108. doi: 10.1186/s12877-020-1503-6 (PMC7076928; doi:10.1186/s12877-020-1503-6)
Supplement: Supplementary file 1 — Additional file 1: Supplementary file 1. Self designed questionnaire Initial English_ENTAiER: Initial Questions. [file 12877_2020_1503_MOESM1_ESM.pdf]

ENTAiER

Initial Questions

Pat.-Nr.

You can ask the study staff to for support when answering the following questions.

Have you fallen in the last 6 months?

Note: If you want to find out what a fall is, you can look up the explanation in the glossary of terms in the patient information.

☐ No

☐ Can't remember

☐ Yes

☐ If so, did you injure yourself so that you needed medical treatment?

☐ No

☐ Yes

☐ Can't remember

Do you use a walking-aid?

☐ No

☐ Sometimes

☐ Always

Please mark with a cross where applicable:

☐ Walking stick ☐ Crutches ☐ Rollator ☐ Wheelchair ☐ Others

Do you have vision problems that are not sufficiently corrected by glasses?

☐ Considerably

☐ Somewhat

☐ No

Do you have hearing problems that restricts you in your daily activities?

☐ Considerably

☐ Somewhat

☐ No

Do you have a vertigo disorder that affects your balance?

☐ Considerably

☐ Somewhat

☐ No

Do you have difficulty walking due to problems in the hips, knees, ankles or in the back?

☐ Considerably

☐ Somewhat

☐ No

Please mark your diseases in the following list.

- ☐ Cardiovascular disease (e.g. High blood pressure, coronary heart disease, peripheral artery disease)
- ☐ Orthostatic syndrome (e.g. vertigo, palpitation or tinnitus when you stand up rapidly)
- ☐ Respiratory disease (e.g. repeated respiratory infections, chronic bronchitis, asthma)
- ☐ Diabetes (Diabetes mellitus)
- ☐ Osteoporosis (bone weakening)
- ☐ Other internal disease (e.g. of the digestive organs, like stomach, liver, intestines; of the kidneys; of the haematopoietic system; of the metabolism, e.g. thyroid gland)
- ☐ Mental distress (e.g. depression, anxiety states)
- ☐ Persistent insomnia or sleep problems
- ☐ Stroke
- ☐ Parkinson's disease
- ☐ Multiple sclerosis
- ☐ Polyneuropathy (e.g. abnormal sensation of the feet, tingling, numbness)
- ☐ Other diseases of the nervous system (e.g. epilepsy, migraine)
- ☐ Joint degeneration (arthrosis) in the hip, knee or ankle joints, which restricts you in your daily activities:
  - ☐ In the knees
  - ☐ In the hips
  - ☐ In the ankle joints
- ☐ Other diseases of the musculoskeletal system (e.g. permanent back pain, rheumatism)

Discuss this list of your illnesses with your study doctor and have him or her sign the following!

Study doctor: I have discussed the diagnoses with the patient.

…………………… ………………………………………………………………

Date Signature study doctor

Do you do any sports (e.g. walking/fast walking, hiking, jogging, gymnastics, tennis, cross-country skiing, faster cycling, fitness studio)?

- ☐ No
- ☐ Once per month
- ☐ Once per week
- ☐ Several times per week

Social activity

How much contact with other people do you have through your living situation?

- ☐ I live alone
- ☐ I live together with my spouse, family member, family or friends
- ☐ I live in a different housing situation (e.g. retirement home, assisted living)

Do you have regular contact with neighbours, friends or family members with whom you do not live in the same household (personal meetings or telephone calls or letters or e-mails)?

- ☐ Less than once per month
- ☐ Several times per month
- ☐ Several times per week

Do you have regular contact with other people, e.g. in a club, music group, religious community, voluntary work, language course, cooking course or similar?

- ☐ Less than once per month
- ☐ Several times per month
- ☐ Several times per week
