# Supplementary material for: A multi-centre, parallel-group, randomised controlled trial to assess the efficacy and safety of eurythmy therapy and tai chi in comparison with standard care in chronically ill elderly patients with increased risk of falling (ENTAiER): a trial protocol
Source: BMC Geriatr. 2020 Mar 17;20:108. doi: 10.1186/s12877-020-1503-6 (PMC7076928; doi:10.1186/s12877-020-1503-6)
Supplement: Supplementary file 2 — Additional file 2: Supplementary file 2. Self designed questionnaire Initial German_ENTAiER: Eingangsfragen. [file 12877_2020_1503_MOESM2_ESM.pdf]

ENTAiER **Eingangsfragen** Pat.-Nr.

Sie können sich bei der Beantwortung der folgenden Fragen vom Studienpersonal unterstützen lassen.

Sind Sie in den letzten 6 Monaten gestürzt?

Hinweis: Wenn Sie nachlesen möchten, was ein Sturz ist, können Sie die Erklärung im Wörterverzeichnis (Glossar) der Patienteninformation nachschlagen.

- ☐ Nein
- ☐ Kann mich nicht erinnern
- ☐ Ja
  - ☐ Falls ja, haben Sie sich dabei verletzt, so dass Sie ärztlich behandelt werden mussten?
    - ☐ Nein
    - ☐ Ja
    - ☐ Kann mich nicht erinnern

Benutzen Sie eine Geh-Hilfe?

- ☐ Nein
- ☐ Manchmal
- ☐ Immer

Bitte kreuzen Sie Zutreffendes an: ☐ Handstock ☐ Unterarm-gehstütze ☐ Rollator ☐ Rollstuhl ☐ Anderes

Haben Sie Sehprobleme, die durch eine Brille nicht ausreichend korrigiert sind?

- ☐ Deutlich
- ☐ Etwas
- ☐ Nein

Haben Sie Probleme beim Hören, die sie bei Ihren Alltagstätigkeiten einschränken?

- ☐ Deutlich
- ☐ Etwas
- ☐ Nein

Haben Sie eine Schwindelerkrankung, die Ihr Gleichgewicht beeinträchtigt?

- ☐ Deutlich
- ☐ Etwas
- ☐ Nein

Haben Sie Schwierigkeiten beim Gehen durch Probleme in den Hüften, Knien, Fußgelenken oder im Rücken?

- ☐ Deutlich
- ☐ Etwas
- ☐ Nein

Kreuzen Sie bitte in der folgenden Liste Ihre Erkrankungen an.

- ☐ Herz-Kreislauferkrankungen (z.B. Bluthochdruck, koronare Herzerkrankung, arterielle Durchblutungsstörungen der Beine)
- ☐ Orthostase-Syndrom (z.B. Schwindel, Herzklopfen oder Ohrensausen beim raschen Aufstehen)
- ☐ Atemwegserkrankung (z.B. wiederholte Atemwegsinfektionen, chronische Bronchitis, Asthma)
- ☐ Zuckerkrankheit (Diabetes mellitus)
- ☐ Osteoporose (Knochenschwund)
- ☐ Andere innere Erkrankung (beispielsweise der Verdauungsorgane, z. B. Magen, Leber, Darm; der Nieren; des blutbildenden Systems; des Stoffwechsels, z.B. Schilddrüse)
- ☐ Psychische Beeinträchtigungen (z.B. Depressionen, Angstzustände)
- ☐ Langandauernde Schlaflosigkeit oder Schlafprobleme
- ☐ Schlaganfall
- ☐ Parkinson (Schüttelkrankheit)
- ☐ Multiple Sklerose
- ☐ Polyneuropathie (z.B. Missempfindungen an den Füßen, Kribbeln, Pelzigkeitsgefühl)
- ☐ Andere Erkrankung des Nervensystems (z.B. Epilepsie, Migräne)
- ☐ Gelenkverschleiß (Arthrose) in der Hüfte, den Knien oder den Fußgelenken, die Sie in Ihren täglichen Aktivitäten einschränkt:
  - ☐ In den Knien
  - ☐ In den Hüften
  - ☐ In den Fußgelenken
- ☐ Andere Erkrankungen des Bewegungsapparats (z.B. dauerhafte Rückenschmerzen, Rheuma)

Besprechen Sie diese Liste Ihrer Erkrankungen mit Ihrem Studienarzt oder Ihrer Studienärztin und lassen Sie ihn oder sie im Folgenden unterschreiben!

Studienarzt/Studienärztin: Ich habe die Diagnosen mit der Patientin oder dem Patienten abgesprochen.

……………………… ………………………………………………………………………….

Datum Unterschrift Studienarzt/Studienärztin

Treiben Sie Sport (z.B. Walking/schnelles Gehen, Wandern, Jogging, Gymnastik, Tennis, Skilanglauf, schnelleres Fahrrad fahren, Fitness Studio)?

- ☐ Nein
- ☐ Einmal im Monat
- ☐ Einmal pro Woche
- ☐ Mehrmals pro Woche

Soziale Aktivität

Wie viel Kontakt zu anderen Menschen haben Sie durch Ihre Wohnsituation?

- ☐ Ich lebe allein
- ☐ Ich lebe zusammen mit Ehepartner, Familienmitglied, Familie oder Freunden
- ☐ Ich lebe in einer anderen Wohnsituation (z.B. Seniorenheim, betreutes Wohnen)

Haben Sie regelmäßigen Kontakt mit Nachbarn, Freunden oder Familienmitgliedern, mit denen Sie nicht im selben Haushalt leben (Persönliche Treffen oder Telefonate oder Briefe oder E-mails)?

- ☐ Weniger als einmal pro Monat
- ☐ Mehrmals pro Monat
- ☐ Mehrmals pro Woche

Haben Sie regelmäßigen Kontakt mit weiteren Personen, z.B. im Verein, Musikgruppe, religiöse Gemeinschaft, ehrenamtliche Tätigkeit, Sprachkurs, Kochkurs oder Ähnliches?

- ☐ Weniger als einmal pro Monat
- ☐ Mehrmals pro Monat
- ☐ Mehrmals pro Woche
