# Supplementary material for: A multi-centre, parallel-group, randomised controlled trial to assess the efficacy and safety of eurythmy therapy and tai chi in comparison with standard care in chronically ill elderly patients with increased risk of falling (ENTAiER): a trial protocol
Source: BMC Geriatr. 2020 Mar 17;20:108. doi: 10.1186/s12877-020-1503-6 (PMC7076928; doi:10.1186/s12877-020-1503-6)
Supplement: Supplementary file 3 — Additional file 3: Supplementary file 3. Self designed questionnaire month 6 English_ENTAiER: Questions About the Last 6 Months. [file 12877_2020_1503_MOESM3_ESM.pdf]

ENTAiER

Questions About the Last 6 Months

Pat.-Nr.

Have you done any balance training in the last 6 months?

- ☐ No
- ☐ Yes
  - ☐ Simple exercises at home (e.g. brushing teeth on one leg)
  - ☐ Tai Chi or Qi Gong (outside of the study)
  - ☐ Dancing
  - ☐ Exercise programme, as recommended in the brochure "Balance & Strength”
  - ☐ Other courses or special training offer
  - ☐ Physiotherapy

Have you done any strength training in the last 6 months?

- ☐ No
- ☐ Yes
  - ☐ Simple exercises at home (e.g. with elastic exercise band)
  - ☐ Sports club or fitness center
  - ☐ Exercise programme, as recommended in the brochure "Balance & Strength”
  - ☐ Other courses or special training offer
  - ☐ Physiotherapy
  - ☐ Regular outdoor exercise (e.g. hiking)

In the last 6 months, have you paid more attention on calcium intake than before? (E.g. through milk, dairy products or in tablet form)

☐ Yes ☐ No ☐ I don’t know

In the last 6 months, have you paid more attention to your vitamin D supply than before? (Staying in the sun, food such as fish, eggs, as a dietary supplement)

☐ Yes ☐ No ☐ I don’t know

ENTAIER

Questions About the Last 6 Months

Pat.-Nr.

Have you paid more attention to safe footwear in the last 6 months than before?

☐ Yes ☐ No ☐ I don't know

Have you made your living environment safer in the last 6 months? (e.g. better lighting, avoidance of tripping hazards or slippery surfaces, railings and handles)

☐ Yes ☐ No ☐ I don't know

Have you visited Internet addresses from the brochure "Balance & Strength" to get ideas for fall prevention?

☐ Yes, many ☐ Only some ☐ No ☐ I don't know

What was helpful for your motivation for the training?

- ☐ Suggestions through the brochure „Balance & Strength“
- ☐ Motivation by study personnel / training group leaders
- ☐ A personal goal that I want to achieve through the exercises
- ☐ The documentation in the training diary
- ☐ I train so as not to fall
- ☐ Exercise manual or DVD from the ENTAIER study
- ☐ Exchange with other study participants
- ☐ Others ______________________________

____

Have you attended Tai Chi classes in the last 6 months (outside the study)?

☐ Yes ☐ No

Have you attended eurythmy courses or eurythmy therapy in the last 6 months (outside the study)?

☐ Yes ☐ No
