# Supplementary material for: A multi-centre, parallel-group, randomised controlled trial to assess the efficacy and safety of eurythmy therapy and tai chi in comparison with standard care in chronically ill elderly patients with increased risk of falling (ENTAiER): a trial protocol
Source: BMC Geriatr. 2020 Mar 17;20:108. doi: 10.1186/s12877-020-1503-6 (PMC7076928; doi:10.1186/s12877-020-1503-6)
Supplement: Supplementary file 4 — Additional file 4: Supplementary file 4. Self designed questionnaire month 6 German_ENTAiER: Fragen zu den letzten 6 Monaten. [file 12877_2020_1503_MOESM4_ESM.pdf]

ENTAiER

Fragen zu den letzten 6 Monaten

Pat.-Nr.

Haben Sie in den letzten 6 Monaten ein Balancetraining durchgeführt?

- ☐ Nein
- ☐ Ja
  - ☐ Einfache Übungen zu Hause (z.B. Zähneputzen auf einem Bein)
  - ☐ Tai Chi oder Qi Gong (außerhalb der Studie)
  - ☐ Tanzen
  - ☐ Übungsprogramm, wie in der Broschüre „Gleichgewicht & Kraft“ empfohlen
  - ☐ Andere Kurse oder spezielles Trainingsangebot
  - ☐ Krankengymnastik

Haben Sie in den letzten 6 Monaten ein Krafttraining durchgeführt?

- ☐ Nein
- ☐ Ja
  - ☐ einfache Übungen zu Hause (z.B. mit Gymnastikband)
  - ☐ Sportverein oder Fitnesscenter
  - ☐ Übungsprogramm, wie in der Broschüre „Gleichgewicht & Kraft“ empfohlen
  - ☐ andere Kurse oder spezielles Trainingsangebot
  - ☐ Krankengymnastik
  - ☐ regelmäßige Bewegung im Freien (z.B. Wandern)

Haben Sie in den letzten 6 Monaten mehr als früher auf die Einnahme von Kalzium geachtet? (z.B. durch Milch, Milchprodukte oder in Tablettenform)

☐ Ja ☐ Nein ☐ Ich weiß nicht

Haben Sie in den letzten 6 Monaten mehr als früher auf die Versorgung mit Vitamin D geachtet? (Aufenthalt in der Sonne, Nahrungsmittel wie Fisch , Eier, als Nahrungsergänzung)

☐ Ja ☐ Nein ☐ Ich weiß nicht

ENTAIER

Fragen zu den letzten 6 Monaten

Pat.-Nr.

Haben Sie in den letzten 6 Monaten mehr als früher auf sicheres Schuhwerk geachtet?

☐ Ja ☐ Nein ☐ Ich weiß nicht

Haben Sie in den letzten 6 Monaten Ihr Wohnumfeld sicherer gestaltet? (z.B. bessere Beleuchtung, Vermeidung von Stolperfallen oder rutschigen Flächen, Geländer und Griffe)

☐ Ja ☐ Nein ☐ Ich weiß nicht

Haben Sie Internet-Adressen aus der Broschüre „Gleichgewicht & Kraft“ besucht, um Ideen zur Sturzprophylaxe zu bekommen?

☐ Ja viele ☐ Nur einzelne ☐ Nein ☐ Ich weiß nicht

Was war für Ihre Motivation zum Training hilfreich?

☐ Anregung durch die Broschüre Gleichgewicht&Kraft

☐ Motivierung durch Studienpersonal / Übungsgruppenleiter

☐ Ein persönliches Ziel, das ich durch die Übungen erreichen möchte

☐ Die Dokumentation im Übungstagebuch

☐ Ich übe, um nicht zu stürzen

☐ Übungsmanual oder DVD aus der ENTAIER Studie

☐ Austausch mit anderen Studienteilnehmern

☐ Sonstiges ____________________

____

Haben Sie in den letzten 6 Monaten (außerhalb der Studie) Tai Chi Kurse besucht?

☐ Ja ☐ Nein

Haben Sie in den letzten 6 Monaten (außerhalb der Studie) Eurythmie Kurse oder Eurythmie-Therapie besucht?

☐ Ja ☐ Nein
