# Supplementary material for: A multi-centre, parallel-group, randomised controlled trial to assess the efficacy and safety of eurythmy therapy and tai chi in comparison with standard care in chronically ill elderly patients with increased risk of falling (ENTAiER): a trial protocol
Source: BMC Geriatr. 2020 Mar 17;20:108. doi: 10.1186/s12877-020-1503-6 (PMC7076928; doi:10.1186/s12877-020-1503-6)
Supplement: Supplementary file 5 — Additional file 5: Supplementary file 5. CTP_V1.1_ENTAiER_20190601: A multi-centre, parallel-group, randomised controlled trial to assess the efficacy and safety of Eurythmy Therapy and Tai Chi in comparison to standard care in chronically ill elderly patients with increased risk of falling (ENTAiER), Version 1.1, 2019-06-01. [file 12877_2020_1503_MOESM5_ESM.pdf]

PROTOCOL

A multi-centre, parallel-group, randomised controlled trial to assess the efficacy and safety of Eurythmy Therapy and Tai Chi in comparison to standard care in chronically ill elderly patients with increased risk of falling

(ENTAiER)

Version 1.1

2019-06-01

Coordinating Investigator
Dr med. Gunver S. Kienle
University Centre for Complementary Medicine. Institute for Infection Prevention and Hospital Epidemiology. Medical Center – University of Freiburg, Germany
Breisacher Str. 115 B, 79106 Freiburg, Germany
Tel. +49 761 270-82010,
Fax: +49 761 270-83230
E-mail: gunver.kienle@uniklinik-freiburg.de

This Protocol contains confidential information. Circulation of this material to individuals who are not involved in the carrying out of the register or any kind of publication requires the approval of the Coordinator. These limitations similarly relate to all confidential information and data which will be obtained in the future.

Approval of the Protocol:

Coordinating Investigator		
Dr Gunver S. Kienle	Signature	Date
Biometrician		
Inga Poguntke	Signature	Date

Investigator Statement

**Title: A multi-centre, parallel-group, randomised controlled trial to assess the efficacy and safety of Eurythmy Therapy and Tai Chi in comparison to standard care in chronically ill elderly patients with increased risk of falling**	
**Study Site:**	
**Principal Investigator at the study site:**	

I confirm that I have read the study protocol and hereby commit myself to adhere to all actions and terms as specified in the relevant sections of the clinical, ethical and general paragraphs.

I confirm that I and my colleagues will abide by the local legislation. I further confirm that the study will be carried out in compliance with the Declaration of Helsinki and the ICH-GCP (R2) guideline.

I acknowledge that all confidential information in this document will not be used or circulated without the prior written consent of the Coordinating Investigator.

Under my supervision I put copies of this study protocol and possible updates as well as access to all information regarding the carrying out of this study protocol at the disposal of my colleagues. I will discuss this study protocol in detail with my colleagues and ensure that they are comprehensively informed about the trial compound/preparation and the execution of the study.

Furthermore I commit myself not to commence patient enrolment before the approval of the responsible ethics committee.

Date (DD/MM/YY) Signature of the Principal Investigator

List of Abbreviations

AE	Adverse Event
ASTS	*Aktuelle Stimmungsskala*
BBS	Berg Balance Scale
BMBF	Bundesministerium für Bildung und Forschung
BMI	Body mass index
BMLSS	Brief Multidimensional Life Satisfaction Scale
CA	competent authority
CI	Confidence Interval
CONSORT	Consolidated Standards of Reporting Trials
CRA	Clinical Research Associate (on-site clinical monitor)
CRF	Case Report Form
CTU	Clinical Trials Unit
DALY	Disability-adjusted life year
DM	Data management
DMC	Data Monitoring Committee
DRKS	German Clinical Trials Register (*Deutsches Register Klinischer Studien*)
eCRF	Electronic case report form
EDC	Electronic Data Capture
EMA	European Medicines Agency
EOT	End Of Treatment
EYT	Eurythmy Therapy
FAS	Full Analysis Set
FIMA	*Fragebogen zur Inanspruchname Medizinischer und Nicht-medizinischer Versorgungsleistungen im Alter*
FPFV	First Patient First Visit
FPI	First Patient In
FRIDS	Fall Risk Increasing Drugs
GDS-15	Geriatric Depression Scale
HR	Hazard Ratio
IADL	Self-Maintaining and Instrumental Activities of Daily Living
ICH-GCP	ICH Topic E6: Guideline for Good Clinical Practice (GCP)
ICPH	Inner Correspondence/Peaceful Harmony with Practices
ICS	Internal Coherence Scale
IEC	Independent Ethics Committee
IRR	Incidence rate ratios
LPI	Last Patient In
LPLV	Last Patient Last Visit
MoCA	Montreal Cognitive Assessment
n	number (mathematically)
NYHA	New York Heart Association
PHI	Protected Health Information
PI	Principal Investigator
PP	Per-protocol set
PSS	Perceived Stress Scale
QOL	Quality of Life Questionnaire
RaR	Rate Ratio

RCT	randomized controlled trial
RR	Risk Ratio
SAF	Safety population
SAP	Statistical Analysis Plan
SAS	Statistical Analysis System
SMD	standardized mean difference
SDV	Source Data Verification
SF-12	Short Form Health Survey, 12 items
Short FES-I	Short Falls Efficacy Scale-International
SOP	Standard Operating Procedure
VAS	visual analogue scale

Contents

Approval of the Protocol: 2

Investigator Statement 3

List of Abbreviations 4

Contents 6

Synopsis 9

Responsibilities 16

1 Introduction 19

1.1 Background and scientific rationale 19

1.2 Evidence 22

1.3 Safety 23

2 Objectives and Endpoints 24

2.1 General considerations, overview 24

2.2 Primary endpoint, definition of falls 25

2.3 Secondary endpoints 26

3 Trial Plan 28

3.1 Trial Design 28

3.2 Treatment arms 28

3.3 Treatment duration 28

3.4 Number of patients 28

3.5 Recruitment rate 28

3.6 Timetable 29

3.7 Participating Study Sites 29

4 Trial Population and Selection Criteria 30

4.1 Inclusion Criteria 31

4.2 Exclusion Criteria 31

5 Recruitment and Enrolment of Subjects in the Study, Randomisation 33

5.1 Patient recruitment 33

5.2 Patient enrolment 33

Pre-screening 33

Waiting time 34

Final Screening, Baseline, Enrolment 34

Start of treatment and observation period 34

5.3 Randomisation – general and methodological considerations ... 35

6 Study Procedures ... 37

6.1 Visit schedule and assessments - Flowchart ... 37

6.2 Study procedures at Study Sites ... 38

6.3 Baseline Assessment: ... 39

6.4 Treatment Phase and Follow-up Assessments ... 41

7 Premature Termination or Suspension of a Study ... 43

7.1 Premature termination of one of the treatment arms or the entire trial ... 43

7.2 Premature termination of the trial at one of the study sites ... 43

7.3 Premature discontinuation of trial participation for individual patients ... 44

7.4 Premature discontinuation of trial treatment for individual patients ... 44

8 Investigational Interventions ... 45

8.1 Intervention description and application ... 45

8.2 Additional interventions ... 47

9 Support of motivation and adherence ... 49

10 Safety monitoring and reporting ... 51

10.1 Complications ... 51

10.1.1 Definition of complications 51

10.1.2 Documentation of complications 52

10.1.3 Assessment of Severity 52

10.2 Serious Complications ... 52

10.2.1 Definition and documentation of serious complications 53

10.2.2 Documentation of serious complications 53

11 Data Monitoring Committee ... 54

12 Data Collection, Handling and Management ... 55

12.1 Data confidentiality ... 55

12.2 Documentation of trial data ... 55

12.3 Data management ... 55

12.4 Data coding 56

13 Quality Assurance System 57

13.1 Quality Control (Clinical Monitoring) 57

13.2 Source Data Verification (SDV) 57

13.3 Quality Assurance (Auditing) 58

14 Biostatistical Planning and Analysis 59

14.1 Study Design 59

14.2 Objectives and Endpoints 59

14.3 Sample Size Calculation 59

14.4 Definition of Populations Included In the Analyses 59

14.5 Methods of Analysis 60

14.5.1 Primary Endpoint 60

14.5.2 Secondary endpoints 61

14.6 Economic Analysis 61

15 Patient Involvement 62

16 Ethical and Legal Principles 65

16.1 Subject Informed Consent 65

16.2 Ethical and Regulatory Requirements 65

16.3 Data Protection and Confidentiality 65

16.4 Archiving 66

17 Registry 67

18 Financing of the project 68

18.1 Financing 68

18.2 Study Reports 68

18.3 Publication of Study Protocol and Results 68

19 References 69

19.1 Relevant Guidelines and Laws 69

19.2 References 70

Synopsis

**TITLE OF TRIAL**	A multi-centre, parallel-group, randomised controlled trial to assess the efficacy and safety of Eurythmy Therapy and Tai Chi in comparison to standard care alone in chronically ill elderly patients with increased risk of falling
**SHORT TITLE**	ENTAiER
**HEALTH CONDITION STUDIED**	Elderly patients with chronic disease and increased risk for falling
**OBJECTIVE(S)**	Primary: To compare Eurythmy Therapy (EYT) plus standard care, Tai Chi plus standard care and standard care alone[1] with respect to the risk of falling over a time period of 6 months.<br><br>Secondary: To compare EYT added to standard care, Tai Chi added to standard care and standard care alone with respect to the number of falls, number of injurious falls, mobility, balance, cognition, mood, quality of life, instrumental activities of daily living, fear of falling, health-care utilization and safety over a time period of 6 and 12 months. To compare EYT, Tai Chi added to standard care and standard care alone with respect to the risk of falling over a time period of 12 months. To compare inner involvement with practices.
**TREATMENTS**	Experimental treatment:<br><br>Intervention 1: EYT exercises twice (week 1-12), later once weekly (week 13-24), in groups, added to standard care (including a brochure with detailed recommendations on fall prevention)<br><br>Intervention 2: Tai Chi exercises twice (week 1-12), later once weekly (week 13-24), in groups, added to standard care (including a brochure with detailed recommendations on fall prevention)<br><br>Control treatment: Standard care alone (including a brochure with detailed recommendations on fall prevention)<br><br>Duration of treatment per patient: 24 weeks (six months)<br><br>Follow-up per patient: six months

[1] for practical purposes the three groups will be referred to in this trial protocol as EYT, Tai Chi, and standard care also, although standard care is present in all three groups

**KEY INCLUSION CRITERIA**	Key inclusion criteria:<br>• Adults 65 years or older.<br>• Chronic disease (musculoskeletal, neurologic, internistic).<br>• Increased risk of falling (Berg Balance Scale (BBS) score 49 or less).<br>• Self-reported history of imbalance.<br>• Living in the community (at own home) or retirement or nursing home.<br>• Ability to participate in 1-hour group-based sessions.<br>• Able to leave home on their own at least twice per week (e.g. for doctors visit, shopping)<br>• Patient's written informed consent has been obtained<br>A specific recruiting strategy will strengthen enrolment of patients which are economically disadvantaged, living a reclusive life.

**KEY EXCLUSION CRITERIA**	Key exclusion criteria:<br>• Medical conditions limiting participation (e.g., terminal illness, heart failure NYHA III-IV, unstable angina pectoris, uncontrolled seizure disorder, decompensated lung disease, cancer in advanced stage, chemo- or radiotherapy ongoing or during last three months, amputation of one or both legs).<br>• Complete dependence on rollator (wheeled walker).<br>• Visual, hearing or language problems affecting understanding of trial documents and procedures.<br>• Major cognitive impairment (MoCA score 18 or less).<br>• High risk for individual support during group session.<br>• Urge incontinence affecting group sessions.<br>• Severe personality disorder, psychiatric disease, alcohol use disorder or other substance use disorders affecting participation of group classes and regular practicing.<br>• Life expectancy of less than 1 year.<br>• Permanent confinement to bed to be expected in less than a year.<br>• Participating in regular Tai Chi or EYT exercises within last 6 months.<br>• Participating in vigorous sports for exercise within last month.<br>• Patient without legal capacity who is unable to understand the nature, significance and consequences of the study.<br>• Participation in a clinical trial within 3 months before the start of this study (screening) or simultaneous participation in another study which could interfere with this study.

**ENDPOINTS**	Primary efficacy endpoint:<br>• Fall (yes/no) within 6 months recorded in diary (i.e. “fallers”).<br><br>Key secondary endpoints:<br>• Number of falls recorded in diary.<br>• Number of injurious falls.<br>• Fear of falling (Short Falls Efficacy Scale-International, Short FES-I).<br>• Mobility and Balance (Berg Balance Scale, BBS).<br>• Cognition (Montreal Cognitive Assessment, MoCA).<br>• Depression (Geriatric Depression Scale, GDS-15).<br>• Health-related quality of life (SF-12).<br>• Self-maintaining and instrumental activities of daily living (IADL).<br>• Inner Correspondence/Peaceful Harmony with Practices (ICPH).<br>• Health Care Utilization: Physician contacts, medication, hospitalizations (FIMA).<br>• use of recommendation of fall prevention brochure.<br><br>Assessment of safety:<br>Complications.
**TRIAL DESIGN**	Multi-centre, randomised controlled trial (1:1:1), stratified by study site, with two active and one control parallel groups.

**STATISTICAL ANALYSIS**	<u>Sample Size:</u><br>Sample size calculation is based on the proportion of patients with at least one fall during 6 months. This proportion is assumed as 50% in the standard care group. The study is planned with 80% power to show a difference between treatments at two-sided level alpha of 5%, when proportions of fallers are 50% with standard care and 35% both with EYT and with Tai Chi. This requires 155 patients per group. To account for a certain amount of non-compliance and incomplete observations, 550 patients will be randomised in total in a ratio of 1:1:1.<br><br><u>Primary efficacy analysis:</u><br>The primary efficacy analysis will be performed in the full analysis set. For the analysis of the primary endpoint fall (yes, no) within 6 months a time-to-event methodology will be applied using the time to the first fall to account for potentially incomplete observation times of the patients. A Cox regression model will be used to test the efficacy of EYT versus Tai Chi versus standard care in reducing the risk of falling within 6 months after handing out the study book. Hazard ratios (HR) between interventions will be calculated from the model with two-sided 95% confidence interval (CI).<br><br><u>Safety:</u><br>Patients will be analysed according to intervention received. Rates of complications will be calculated with two-sided 95% CI.<br><br><u>Secondary endpoints:</u><br>Secondary endpoints will be analysed with appropriate regression models.	
**SAMPLE SIZE**	To be assessed for eligibility:	n = 2000 per phone, 1000 personally
	To be enrolled and randomised:	n = 550
	To be analysed:	n = 550
**TRIAL DURATION**	Recruitment period (months):	24
	First patient in to last patient out (months):	36
	Treatment duration per patient (months):	6
	Follow-up duration per patient (months):	6
**PLANNED DATES**	Enrolment of first patient, first patient in (FPI)	3rd quarter 2019

	Enrolment of last patient, last patient in (LPI)	2nd quarter 2021
	End of trial for last patient, last patient last visit (LPLV)	2nd quarter 2022
	Final statistical analysis	4th quarter 2022
**PARTICIPATING SITES**	8 study sites in Germany	
**FUNDER**	Federal Ministry of Education and Research (BMBF 01GL1805)	

Study Flow Chart

Pre-Screening
per phone or personal screening (n=2,000)

Waiting time
until n~30 patients at study site are potentially eligible

Screening and Baseline
Inclusion/exclusion criteria, informed consent (n=1,000, at study site n~30),
Baseline (n~550; at study site n=12, 15 or 18)

Randomisation
Allocation 1:1:1 (n=550, at study site n=12, 15 or 18)

Handing out study book: start of observation

**EYT** (6 months)<br>in groups n=5 (+/- 1)<br>added to standard care	**Tai Chi** (6 months)<br>in groups n=5 (+/- 1)<br>added to standard care	**Standard Care** (6 months)<br>Recommendation to<br>primary care doctor's visit

Follow-up (6 months)

Analysis, Publication

Responsibilities

**"Sponsor"** (according to BMBF)	Institution:<br><br><br>Address:	**Medical Center - University of Freiburg**<br>represented by the Executive Medical Director<br>(Leitender Ärztlicher Direktor)<br>Breisacher Str. 153<br>79106 Freiburg, Germany
**Coordinating Investigator**	Name:<br>Institution:<br><br><br><br>Address:<br>Telephone:<br>Fax:<br>E-mail:	**Dr med. Gunver S. Kienle**<br>University Centre for Complementary Medicine. Institute for Infection Prevention and Hospital Epidemiology. Medical Center – University of Freiburg, Faculty of Medicine, University of Freiburg, Germany<br>Breisacher Str. 115 B, 79106 Freiburg, Germany<br>+49 761 270-82010<br>+49 761 270-83230<br>gunver.kienle@uniklinik-freiburg.de
**Medical trial coordinator**	Name:<br>Institution:<br><br><br><br>Address:<br>Telephone:<br>Fax:<br>E-mail:	**Dr med. Paul Werthmann**<br>University Centre for Complementary Medicine. Institute for Infection Prevention and Hospital Epidemiology, Medical Center – University of Freiburg, Faculty of Medicine, University of Freiburg, Germany<br>Breisacher Str. 115 B, 79106 Freiburg, Germany<br>+49 761 270-83200<br>+49 761 270-83230<br>paul.werthmann@uniklinik-freiburg.de
**Biostatistician**	Name:<br>Institution:<br>Address:<br>Telephone:<br>Fax:<br>E-mail:	**Inga Poguntke**<br>Clinical Trials Unit, Medical Center – University of Freiburg<br>Elsaesser Str. 2, 79110 Freiburg, Germany<br>+49 761 270-77107<br>+49 761 270-73770<br>inga.poguntke@uniklinik-freiburg.de
**Randomisation**	Institution:<br>Address:<br>Fax:	Clinical Trials Unit<br>Elsaesser Str. 2, 79110 Freiburg, Germany<br>+49 761 270-74390
**Project Manager**	Name:<br><br>Institution:<br>Address:<br>Telephone:<br>Fax:<br>E-mail:	**Dr phil. Birgit Grotejohann**<br>Clinical Trials Unit<br>Elsaesser Str. 2, 79110 Freiburg, Germany<br>+49 761 270-74000<br>+49 761 270-73770<br>Clinical Trials Unit Freiburg<br>birgit.grotejohann@uniklinik-freiburg.de
**Monitoring (CRA(s))**	Institution:<br>Address:	Clinical Trials Unit<br>Elsaesser Str. 2, 79110 Freiburg, Germany
**Data management**	Institution:<br>Address:	Clinical Trials Unit<br>Elsaesser Str. 2, 79110 Freiburg, Germany
**Scientist**	Name:	**Dr rer. pol. Klaus Kaier**

	Institution:	Institute of Medical Biometry and Statistics, Medical Center—University of Freiburg
	Address:	FDM, Ernst-Zermelo-Str. 1, 79104 Freiburg, Germany
	Telephone:	+49 761 203-6807
	Fax:	+49 761 203-7700
	E-mail:	kaier@imbi.uni-freiburg.de
**Data Monitoring Committee**	Name:	**Prof Dr Peter Wayne**
	Institution:	Osher Center for Integrative Medicine, Harvard Medical School, Brigham and Women's Hospital, Boston
	Address:	Brigham and Women's Hospital<br>Preventive Medicine<br>900 Commonwealth Ave<br>Boston MA 02215, USA
	Name:	**Prof Dr med. Klaus Linde**
	Institution:	Institut für Allgemeinmedizin, Technische Universität München, Medizinische Fakultät, München
	Address:	Orleansstraße 47<br>81667 München, Germany
	Name:	**Dr phil., Dipl.-Math. Marietta Kirchner, M.A.**
	Institution:	Institute for Medical Biometry
	Address:	Im Neuenheimer Feld 130.3<br>69120 Heidelberg , Germany
	Name:	**Prof Dr Gene Feder**
	Institution:	Centre for Academic Primary Care, School of Social and Community Medicine, University of Bristol
	Address:	Office Room 1.01c<br>Canynge Hall,<br>39 Whatley Road, Bristol BS8 2PS , UK
**Advisory Board**	Name:	Geriatric Counselling<br>**Dr med. Bernhard Heimbach, Dr Sebastian Voigt-Radloff**
	Institution:	Centre for Geriatric Medicine and Gerontology (ZGGF), Medical Center – University of Freiburg, Faculty of Medicine, Germany
	Address:	Lehener Strasse 88<br>79106 Freiburg, Germany
	Name:	Representatives of patients and public:<br>**Senior Council of the City (Stadtseniorenrat Freiburg e.V.),**
	Address:	Schusterstraße 19<br>79098 Freiburg, Germany
	Name:	**Sabine Ringer, Beate Saur**
	Address:	Haus Morgenstern<br>Gänsheidestraße 100,<br>70186 Stuttgart, Germany
	Name:	**Karl-Hermann Lieberknecht**
	Address:	Am Bruckwald 1<br>79183 Waldkirch, Germany
	Name:	Tai Chi Counselling<br>**Axel Dreyer**

Institution:	Taiji Schule Axel Dreyer
Address:	Innsbruckerstr.13<br>79111 Freiburg, Germany
Name:	**Markus Maria Wagner M.A.**
Institution:	Bundesvereinigung für Taijiquan und Qigong (BVTQ)
Adress:	Taiji-Akademie<br>Emil-Mannkopffstr. 6<br>35037 Marburg, Germany
Name:	**Dr rer. medic. Christoph Stumpe**
Institution:	Deutscher Dachverband für Qigong und Taijiquan e. V. (DDQT)
Adress:	Shen Men Institut<br>Hüttenstraße 31 a<br>40215 Düsseldorf, Germany
Name:	EYT Counselling<br>**Isabel Martin, Angelika Wendt**
Address:	Starkenstr. 36<br>79104 Freiburg, Germany

1 Introduction

1.1 Background and scientific rationale

Age is strongly associated with an increased risk of chronic diseases, multi-morbidity, polypharmacy and impaired sensory, movement and cognitive functions. This increases suffering, immobility, frailty, risk of falling, dependence and health-care utilization [1-3]. In a vicious circle, physically inactive lifestyle, social isolation, reduced autonomy and particularly falls increase the risk for morbidity and mortality [2-6]. To interrupt this vicious circle and to enable healthy aging despite chronic disease, it is essential to reduce risk and fear of falling and to enhance physical, psychological and cognitive capacities [1-3]. For this purpose, integrative medical methods, mind-body interventions like Tai Chi [7-10] are used in older people [11]. Another mind-body oriented exercise therapy is EYT [12, 13], training body movements as well as concentrative, sensory and mindfulness capacities. Daily mind-body oriented exercises with sensory and concentrative elements are expected to bring more awareness for slower and safer movements while performing activities of daily living. These activities are often dual tasks (e.g. moving and carrying) and can be negatively influenced by stress or fear of falling. Repetitive EYT exercises may support the ‘automatization’ to slow and safe body movements. EYT aims at activation of patients, enhancing balance, mobility, self-perception and physical fitness, and strengthening salutogenetic resources. EYT does not add to polypharmacy.

80% of older adults have at least one chronic disease and 65% have at least two [14, 15]. The incidence rate of falls of Germans aged 65-90 is 0.3-0.4 (men and women) per person/year [16], and increases with chronic diseases (up to 3 in Parkinson disease [17]). The incidence rate of “recurrent falls” is 0.1 per person/year [16]. 10-20% of falls lead to injuries, 5% to fractures, 1-2% to femoral neck fractures [3]. Prevalence of fear of falling in older adults ranges from 21% to 85% [18]. Up to 30% of people after femoral neck fractures and up to 50% of people in high age treated in hospital due to a fall, die within one year [3].

Risk factors for falls include [19]: age, female gender, past history of a fall, lower-extremity weakness, cognitive impairment, loss of sensory system functions (e.g. proprioceptive sensitivity, vestibular system, vision, hearing), Parkinson disease, osteoarthritis of the knee, diabetes, cerebrovascular or cardiovascular disease, history of stroke, orthostatic hypotension, balance problems, dizziness, anaemia, drugs affecting the central nervous system, number of medications of any type, recent changes in the dose of medication, type of footwear and safety hazards in the home environment.

Disability-adjusted life year (DALY) rates caused by falls are 5,000-7,000 per 100,000 men and women aged 80 and older [20]. After femoral neck fractures only 33-40% of patients regain their prior basic everyday competences (eating, body care) and 14-21% their competence for instrumental activities (like shopping, phone calls). After falls and hip fracture, 30-50% of people express fear of falling reducing physical activity, mobility, self-confidence and increasing falls, social isolation, institutionalization, and mortality [3, 21].

Novelty:

Chronic disease, multi-morbidity and risk of falls are complex and multifactorial. Most health care systems do not provide comprehensive care and most guidelines focus on just one disease, thus increasing the risk for polypharmacy [2]. Recommended for fall reduction and mobility are educational programs, training of strength and balance, treating osteoporosis, optimization of medications, vision, shoes, and environmental factors [22-24]. However, elder people with increased risk of falling often do not use recommended exercise training. Only 14% of people aged 70-79 are physically active as recommended (at least 2.5 hours per week) [25]. Reasons to avoid exercise are health issues, reduced well-being, negative experience, but also experiencing exercise like a 'chore', a 'big deal'. Structured 'artificial' activities, such as using an exercise bicycle had negative connotations. Supportive for regular exercise are an appreciating, encouraging social context, indoor activity, achievable instead of 'hard work', starting with minimal-intensity, experimenting individually and exercises that are interesting, safe and pleasurable, with noticeable improvements (mood, confidence, energy), and also the self-perceived risk of falls and injuries [26-28].

Tai Chi for fall prevention has been widely evaluated (see section 1.2). EYT is similar to Tai Chi, they pursue comparable multicomponent movements, simple or complex, smoothly flowing, creating sensation and awareness of limb movement and balance. They address important fall risk factors: strength, balance, coordination, postural control, mobility, and fear of falling [29]. Both include similar "Active Ingredients": Awareness, Intention, Structural Integration, Active Relaxation, Strengthening and Flexibility, Natural, Freer Breathing, Social Support, and Embodied Spirituality [10]. EYT, and partly also Tai Chi, teaches patients to integrate with every movement a concentrated perception of other body parts and the body as a whole, to bring awareness to balance, movement, position, tension of body parts, and perceive warmth, light, tightness, wideness and space around. Mindfulness is achieved through the calm and concentrative setting.

Potential advantages of EYT are: While Tai Chi is rooted in eastern, EYT is rooted in western culture and philosophy. This may have an impact on preferences of the patients. EYT movements are intimately linked to music, phonems, poems or rhythms from the western cultural and emotional background, beyond giving instructions. This increases the memorability of exercises, they can be easier practiced alone despite their inherent complexity; it also links to positive emotional and familiar issues and to art. The intense focus on various sensory capacities may be stronger. EYT can easily adapt to patients with major functional limitations. While Tai Chi has its focus primarily in the preventive, outpatient and in the rehabilitative setting, EYT is also established in the inpatient secondary and tertiary care setting, including bedridden and intensive care patients, additional to the outpatient and rehabilitative context. The duration of training is longer in EYT (at least 5.5 years). – Therefore, while direct effects on falls are expected to be similar, differences are expected with regard to preferences, identification with the intervention, memorability of exercises, mood, quality of life, and potentially in better adaption to increased physical constraints. – Altogether, patients are highly satisfied with EYT

[30-32], which is supported by the treatment context, group setting, no precondition of physical fitness and avoiding negative connotations.

As the importance and popularity of integrative and mind-body-based interventions have significantly increased during the last decades, particularly among people entering the age of 60+ and 70+ and 80+ within the next years [33-35], an increasing amount of people in this age population can be expected to have a strong preference for integrative mind-body interventions, compared to mere physical training of strength and balance. The proportion of the US American population using Yoga, Tai Chi and Qigong has nearly doubled in 10 year, reaching nearly 10%, and increases also in people aged ≥ 65 [33]. As motivation and availability decide upon actually training, an assessment of the impact of these interventions on health outcomes and inner involvement in chronically ill patients with increased risk of falling is of major importance.

Clinical impact:

Reducing risk of falling and fear of falling and improving balance, mobility, functional independence, quality of life, mood and cognition support the management of chronic diseases, physical fitness and "healthy" aging.

Patient benefit:

Falls with injuries are serious events in older patients' biographies and often mark the end of their independence [15]. 80% of women aged 75 or older preferred death to a „bad" hip fracture resulting in loss of independence and nursing home admission [36]. Falls, also non-injurious, lead to a decline of social activities, mobility, and cognitive and physical performance in older people [37, 38]. Therefore a reduction of falls would be of significant benefit for patients, as well as reduced fear of falling, improved mobility, self-perception, independence, mood and cognition.

Socioeconomic impact:

Reduced physical activity is a strong predictor of health care utilization (medications, physician contacts), costs, morbidity and mortality [1, 3]. In Germany, 350.000 or more hospitalizations of people aged 65 or more were due to falls in 2006 [3]. In European countries, North America, and Australia, 0.85% to 1.5% of the total national health care expenditures are spent on fall-related costs, which correspond to 0.07% to 0.20% of the GDP [39]. Reducing the risk of falls, fear of falling, and increasing mobility, activity, socialization and mood may therefore reduce morbidity, costs and healthcare utilization.

1.2 Evidence

On average, effects of EYT directly on falls, gait and balance are expected to be comparable with Tai Chi (see “Novelty”, 1.1).

Mind-body interventions: According to a Cochrane review, Tai Chi reduced the rate of falls (rate ratio (RaR) 0.72, 95% CI 0.52-1.00; 5 trials) and the risk of falling, i.e. the number of people experiencing one or more falls (risk ratio (RR) 0.71, [95%CI 0.57-0.87]; 6 trials). [7] This is supported by a recent Cochrane review reporting a reduction of rate of falls of 19% with Tai Chi (RaR 0.81, 95% CI 0.67 to 0.99; 7 trials) as well as risk of falling by 20% (RR 0.80, 95% CI 0.70 to 0.91; 8 trials). All types of exercises reduced the rate of falls by 23% (rate ratio (RaR) 0.77, 95% confidence interval 0.71 to 0.83; 59 trials) and the risk of falls by 15% (risk ratio (RR) 0.85, 95% CI 0.81 to 0.89; 63 trials). Exercises may also reduce the number of people experiencing one or more fall-related fractures (RR 0.73, 95% CI 0.56 to 0.95, 10 trials, low-certainty evidence) and the number of people experiencing one or more falls requiring medical attention (RR 0.61, 95% CI 0.47 to 0.79; 5 trials), however, the evidence is less certain. Particularly important seem to be exercise programmes that involve balance and functional exercises.[40] Falls decreased also after dancing and music based training. Fear of falling significantly improved with Tai Chi, yoga, training of balance, strength and resistance (SMD 0.37, 95% CI 0.18-0.56) [8], as well as balance [9].

Repeating exercises under instruction and close observation is important for learning, for training, for individually tailoring the exercises to the capabilities of the patients. Biweekly or weekly instructed group sessions for an extended time is routine in Tai Chi classes and has also been practiced in other Tai Chi trials. Tai Chi intervention should involve at least 50h of practice, to be effective [41].

Eurythmy Therapy (EYT): The Austrian *Allgemeine Unfallversicherungsanstalt* described a reduction of accidents from prior constant 5% to zero, and a reduction of sick leave in construction workers after implementation of EYT as part of a training and counselling program [42]. In a multi-centre clinical cohort study, EYT was prescribed to 419 chronically ill outpatients (31.7% of patients with mental disorders 23.4% with musculoskeletal diseases), with a median disease duration of 3.0 years: Disease severity improved and quality of life increased within three months and were maintained for 24-months, while health-care utilization (physician contacts, number of drugs) decreased [30]. A recent randomised controlled trial comparing EYT, yoga and physiotherapy in 270 patients with chronic back pain, found a comparable improvement of pain (VAS) and functional capacity (self-rated physical disability caused by low back pain, RMDS, SF-12 physical component) in all groups, but under EYT (partly also under yoga, not though under exercise therapy) it found a significantly larger improvement in mental component of quality of life (SF-12) and a significant improvement of Inner Correspondence/Peaceful Harmony with Practices (ICPH), internal coherence (ICS), stress perception (PSS), mood (ASTS) and life satisfaction (BMLSS) (Büssing et al. manuscript in preparation, DRKS00004651). An improvement of initially pronounced deficits in cognitive and neuromotor functions and visuomotor integration was found in 7 paediatric cerebellar tumour

survivors under EYT [31]. Cancer-related fatigue in breast cancer survivors improved with EYT as part of a multimodal program (sleep education, psycho-education, eurythmy- and painting-therapy), comparable or larger than with aerobic training [43]. Heart rate variability showed an improved autonomic regulation, a change from a risky stress coping pattern to a healthier pattern, associated with an improved quality of life (e.g. [44, 45]). – EYT had been safe.

1.3 Safety

EYT and Tai Chi pursue gentle, smoothly flowing movements. Their risk can be assumed to be comparable to normal physical exercise applied to reduce fall risk. Trials investigating exercises for preventing falls in elderly in general report minor harms, including pain, bruising or fall injuries or fractures that occurred during the exercise session. Trials comparing exercise intervention with a control group reported no difference in the rate of serious injuries [40, 46]. No specific risk is associated with EYT and Tai Chi; they are unlikely to result in serious adverse events, but may be associated with minor musculoskeletal aches and pains or other side effects of gentle exercises. One extensive systematic review on safety of Tai Chi identified 153 RCTs, (searched through March 2013), most targeting older adults. Of these, only 50 had included reporting of AEs, and only 18 trials (12% overall) also reported an explicit AE monitoring protocol. Reported AEs were typically minor and expected, and primarily musculoskeletal related (e.g., knee and back pain); no intervention-related serious AEs were reported [47]. Cochrane Reviews on Tai Chi for preventing falls and reducing fear of falling on older people had not identified any risk [7, 8, 40]. A recently published trial on Tai Chi in preventing falls among older adults at high risk of falling had documented 2 falls during classes in 85 community-dwelling adults 70 years or older during 48 60-minute Tai Chi classes. Altogether, in this trial, Tai Chi had reduced the risk of falling [48].

Regarding EYT, no specific side effects are known. In an observational study without a control group, 3.1% (13/419) of patients receiving EYT had noted unspecific adverse reactions to EYT (like symptom aggravation, inner tension, depressed mood) during a period of 24 months. The study team had not assessed a causal relationship of these patient-reported adverse reactions to EYT. None of the patients had stopped EYT due to the adverse reactions [30]. A trial investigating a combined multimodal intervention including EYT in 126 breast cancer survivors with chronic cancer-related fatigue over a 10-week period (140–165 minutes per week) found 87 possible adverse therapy reactions to the multimodal intervention, like ‘back pain’, ‘dizziness during eurythmy’; ‘increasing exhaustion through sleep restriction/stimulus control’ [43], Beyond, no specific risks are known [12, 13].

2 Objectives and Endpoints

2.1 General considerations, overview

The main objective of the trial is, whether EYT and Tai Chi can interrupt the vicious circle of chronic disease, falls, inactivity, loss of independence, reduced quality of life (see section 1.1). The primary question thereby is, whether EYT (and Tai Chi) can reduce risk of falling (fallers), which often mark the beginning of dependency and decline of mobility. Since about 50% of the patients are expected to fall with standard care, this is considered the mainly relevant endpoint and therefore chosen as primary. The number of falls is chosen as secondary endpoint. – The next important question is, whether EYT (and Tai Chi) can improve mobility and balance and reduce injuries through falls and fear of falling, which are often the primary threat to mobility. The BBS, number of injurious falls and falls efficacy scale assesses these as secondary outcome parameters.

The next question is, whether this leads to better management of daily tasks, which will be assessed by the IADL. As the overall objective is better coping with chronic disease, enabling healthy aging despite chronic disease, the next questions refers to changes in health related quality of life, cognition and mood. These are assessed with SF-12, MoCA and GDS-15. FIMA will be used to estimate an impact on health-care utilization. This will also enable economic evaluations. Finally, the ICPH will assess in how far the patients identify with their intervention.

Objectives and related endpoints (assessed at month 0, 3, 6, 12 after handing out the study book)

	**Objective**	**Endpoint**
**Primary**	Can EYT and Tai Chi reduce risk of falling within 6 months?	Risk of falling: incidence of experiencing at least one fall within 6 months (“fallers”)
**Secondary**	Can EYT and Tai Chi<br>1. reduce the risk of falling within 12 months?<br>2. reduce the numbers of falls?<br>3. reduce the injuries through falling?<br>4. reduce fear of falling?<br>5. improve mobility and balance?<br>6. improve cognition?<br>7. improve mood?	1. The primary endpoint risk of falling will be evaluated at 12 months<br>2. Number of falls in the groups<br>3. Number of injurious falls<br>4. Short Falls Efficacy Scale-International (Short FES-I) [49]<br>5. Berg Balance Scale (BBS) [50]<br>6. Montreal Cognitive Assessment (MoCA) [51]

	Objective	Endpoint
	8. improve health-related quality of life?<br>9. improve management of daily tasks?<br>10. induce identification and agreement with the intervention?<br>11. impact health-care utilization?<br>12. use of fall prevention recommendations. Estimation of motivational support. Tai Chi, EYT contamination in comparison groups.<br>13. Safety: Do EYT and Tai Chi induce harm?<br>14. Compliance	7. Geriatric Depression Scale (GDS-15) [52]<br>8. Health-related quality of life (SF-12) [53]<br>9. Self-maintaining and instrumental activities of daily living (IADL) [54]<br>10. Inner Correspondence/Peaceful Harmony with Practices (ICPH) [55]<br>11. *Fragebogen zur Inanspruchnahme medizinischer und nicht-medizinischer Versorgungsleistungen im Alter* (FIMA) [56]*<br>12. Self-designed questionnaire<br>13. Complications<br>14. Number of participated group sessions and number of days practiced at home

* reduced to questions of doctors and specialists visits, therapies, inpatient or outpatient care, hospitalization, rehabilitation, surgery, medical aids, place of living.

2.2 Primary endpoint, definition of falls

The primary endpoint is chosen as the risk of falling, i.e. the fact if a patient falls at least once during a 6 months period or not. The time to first fall is used in the statistical analysis, calculated as time from handing out the study book to the first fall. For patients who do not fall within the 6 months observation period, the time to last contact will be used as censored observation. Death without previous fall will be regarded as competing event in the analysis. The secondary outcome number of falls is calculated as the number of falls recorded in the diary and ascertained by telephone visits. This will be analysed in relation to the individual follow-up time of the patients.

Definition, classification

A fall is defined as “an unexpected event in which the participants come to rest on the ground, floor or lower level” [57].

An event is classified as a fall when the patient by accident, unintentionally lands on the floor or the ground, or fall and hit objects like stairs or pieces of furniture on a lower level. It is a rapid change from the upright/sitting position to the reclining or almost lengthened position [48, 57].

An event is not classified as a fall, if

- it is an intentional change to position to rest in furniture, wall or other objects, like lying down, sitting down, kneeing down;
- it is due to a syncope, seizure or another condition causing unconsciousness;
- it is a consequence of sustaining a violent blow or other violent impact;
- it is due to a sudden onset of paralysis as in stroke or an epileptic seizure;
- it is a sudden event of dizziness, uneasiness, vertigo or bumping into objects (e.g. furniture), without being accompanied by a fall (as defined above).

Frequency of falls will be self-reported daily in fall diaries and ascertained in monthly visits. Its assessments follows international guidelines [57].

2.3 Secondary endpoints

Risk of falling at 12 months will be defined as in 2.2 within 12 months.

Injurious falls (total number) are subdivided by “moderate” and “serious” [48]:

1) Moderate injurious falls are defined as those that resulted in sprains, bruises, scrapes, or joint injuries or if the individual needed medical care

2) Serious injurious falls are defined as those that resulted in a fracture, head injury, tendon rupture, or admission to a hospital (inpatient care), or that required stitches.

The BBS is a 14-item scale primarily assessing standing balance and sit-to-stand positions. Scores are based on a 5-point scale, rating from 0 to 4, based on performance quality and duration, or assistance needed. “0” indicates the lowest and “4” the highest level of function. The total score ranges from 0 to 56 points [50].

The MoCA is a brief cognitive screening tool for mild cognitive impairment and assesses several cognitive domains. The total sum score ranges from 0 to 30 and higher scores indicate higher functioning [51].

The Short FES-I is a 7-item questionnaire to measure fear of falling in elderly patients during different activities. It is to be filled out by the patient him/herself. Each item is rated on a four-point scale from 1 (not at all concerned) to 4 (very concerned). The total score ranges from 7 (no concern about falling) to 28 (severe concern about falling) [49].

The GDS-15 is a short, 15-item instrument, developed and validated for measurement of depressive symptoms among elderly people. It is to be filled out by the patient him/herself. Its items require a yes/no response. The total score ranges from 0 to 15. Sum scores 0-4 are considered normal, sum scores 12-15 indicate severe depression, in between as mildly depressed [52].

The SF-12 is a short-form generic measure of health status from the patient’s point of view. It is to be filled out by the patient him/herself. It contains 12 items on 8 health concepts. Results are expressed in terms of two meta-scores: Physical Component Summary and Mental Component Summary. Scores range from 0 to 100. Higher scores indicate better functioning. The mean score in a representative sample of the US population is 50 [53].

The IADL evaluates complex instrumental activities of daily living, on 8 items. The total score ranges from 0 to 8 in women, and from 0 to 5 in men [54].

The ICPH is a questionnaire to measure the Inner Correspondence/Peaceful Harmony with Practices (ICPH) of individuals with mind-body interventions. It is to be filled out by the patient him/herself. It contains 12 items, which are scored on a 5-point scale from disagreement to agreement. The total score ranges from 0 to 60. Scores >50 indicate higher agreement (positive attitude), scores <50 indicate disagreement (negative attitude) [55]. Only groups EYT and Tai Chi will perform this questionnaire.

FIMA is a generic questionnaire for the use of medical and non-medical services in old ages. It is to be filled out by the patient him/herself.

Medications are documented by the investigator. They are restricted to total number of drugs and to classes of fall risk increasing drugs (FRIDs), according to the list from the Swedish National Board of Health and Welfare[2]:

- (increase fall risk) Opioids, Antipsychotics (lithium excluded), Anxiolytics, Hypnotics and sedatives, Antidepressants
- (may cause of worsen orthostatism) Vasodilators used in cardiac diseases, Antihypertensives, Diuretics, Beta blocking agents, Calcium channel blockers, Renin-angiotensin system inhibitors, Alpha-adrenoceptor antagonists, Dopaminergic agents, Antipsychotics (lithium excluded) Antidepressants

Herbal preparations, vitamins or homeopathic medicinal products are excluded.

Use of standard fall prevention measures, subjective estimation of motivational support; contamination with Tai Chi or EYT in non-Tai Chi and non-EYT groups, will be asked at month 6 only, using a self-designed questionnaire.

Compliance:

Compliance with group sessions is defined as participating in at least 70% of the group sessions.

Compliance with practicing at home is defined as practicing on average at least 70% of the recommended three times per week, irrespective of the duration.

Safety:

For details on definition and mode of analysis of complications see section 10.1.

[2] Fastbom J, Schmidt I. Indikatorer för god läkemedelsterapi hos äldre. The Swedish National Board for Health and Welfare 2010 [http://www.socialstyrelsen.se/publikationer2010/2010-6-29]

3 Trial Plan

3.1 Trial Design

Multi-centre, randomised controlled, open-label trial (1:1:1), stratified randomised by study site, with two active and one control parallel groups. The objective is to compare EYT, Tai Chi (each added to standard care[3]) and standard care alone in elderly patients with chronic disease and increased risk for falling with respect to the risk of falling over a time period of 6 months (primary objective) and with respect to the number of falls, injurious falls, mobility, cognition, mood, quality of life, instrumental activities of daily living, fear of falling, health-care utilization, agreement with practices, use of fall prevention recommendations, estimation of motivational support, Tai Chi, EYT contamination in comparison groups and safety over a time period of 3, 6 and 12 months (secondary objectives).

3.2 Treatment arms

Patients will be allocated 1:1:1 to EYT arm or Tai Chi arm or standard care alone arm (control). For details on treatment please refer to section 8.

3.3 Treatment duration

Patients will be treated with EYT or Tai Chi for 6 months (month 1-3 twice per week, month 4-6 once per week). For details of premature termination see section 7. Standard care has no time limitation.

3.4 Number of patients

A total of 550 patients will be randomised.

3.5 Recruitment rate

The average recruitment rate is approximately 75 patient /quarter, starting quarter III 2019.

The recruitment capacity of the study sites for the complete trial is

**Affiliation**	**Expected number of patients recruited for the complete trial**
Freiburg University Hospital, Centre for Complementary Medicine	≥ 200

[3] for practical purposes the three groups will be referred to in this trial protocol as EYT, Tai Chi, and standard care alone, although standard care is present in all three groups

Tübingen University, Institute of General Practice and Interprofessional Care	100
University of Witten Herdecke	≥ 60
University of Essen, Klinik für Naturheilkunde und Integrative Medizin	≥ 60
Immanuel Hospital Berlin, Department of Internal and Complementary Medicine; Charité Berlin, Institute for Social Medicine, Epidemiology and Health Economics	60
Havelhöhe Hospital, Geriatrics, Berlin	45
Filderklinik, ARCIM Institute	30
Integrative Medicine, Ulm University Hospital	30

3.6 Timetable

Enrolment of first patient (FPFV)	3rd quarter 2019
Enrolment of last patient	2nd quarter 2021
End of trial for last patient (LPLV)	2nd quarter 2022
Final statistical analysis	4th quarter 2022
Treatment duration per patient	6 months
Planned interim analysis	None

3.7 Participating Study Sites

Eight study sites are planned in Germany, which must meet the structural and personnel requirements for performing the planned regular study-related investigations.

If necessary, additional qualified study sites can be included in the performance of the study.

4 Trial Population and Selection Criteria

Trial population will be elderly patients with chronic disease and increased risk for falling.

A specific recruiting strategy will strengthen enrolment of patients which are economically disadvantaged, living a reclusive life.

Inclusion criteria are broad, reflecting the complex situation of chronic diseases and falls in aging patients and ensuring feasibility of recruitment and generalizability of the results. The criteria ensure to enrol patients in real need of therapy (i.e. clear increased fall risk and mobility impairment), but still having enough mobility to pursue the exercises and attend the classes and understand the logistics of the trial; the inclusion criteria also leave room for improvement of the condition if the intervention is successful:

The BBS, being a key inclusion criteria, is widely used to determine a patient's mobility and balance during a series of predetermined tasks [50] and is an appropriate tool to predict falls at a moderate accuracy level [58]. A BBS score of 49 combined with self-reported history of imbalance predicts a 50% risk of falls (2 falls within 6 months), with a specificity of 82% and a sensitivity of 91% [59]. That means, that a patient population with a BBS score of 49 or less (and history of imbalance) will have an at least 50% risk of falling within the next 6 months, indicating a clearly increased risk of falls [50, 59] and consecutively an indication for an intervention. This also refers to the "younger" part of the population of adults aged ≥ 65 years, as they can have an increased fall risk due to a chronic disease. A BBS score of 39 or less indicates a fall risk above 90%. In the range 54 to 46 a 1-point change in the BBS scores led to a 6% to 8% increase in fall risk [59]. Residents of a home for elderly that had an average BBS score of 48.3 (95% CI 47-49.6) used cane outdoors; elderly that had an average score of 45.3 (95% CI 44-46.5) used a cane also indoors [50].

Exclusion criteria shall ensure participation, the attendance of group sessions and follow-up visits: – "Visual, hearing or language problems affecting understanding of trial documents and procedures" will exclude patients that cannot comply with all trial requirements, understand informed consent, understand questionnaires, tests, diary documentation, etc. – "Medical conditions limiting participation", "Complete dependence on rollator", "Major cognitive impairment", "Severe personality disorder or psychiatric disease affecting group classes" will exclude patients who cannot participate in or benefit from group exercise classes. – "Life expectancy of less than 1 year" and "Permanent confinement to bed to be expected in less than a year" will exclude patients not to be expected to complete the trial. – "Participating in vigorous sports for exercise within last month" will exclude people already highly active, and less in need for an intervention. – "Participating in regular Tai Chi or EYT exercises currently or during the last 6 months" will exclude patients that will probably not benefit from the intervention as they already received it.

4.1 Inclusion Criteria

- Adults 65 years or older.
- Chronic disease (musculoskeletal, neurologic, internistic)
- Increased risk of falling (Berg Balance Scale (BBS) score 49 or less).
- Self-reported history of imbalance (*Anamnestisch: Unsicherheit beim Gehen oder Stehen*)
- Living in the community (at own home) or retirement or nursing home.
- Ability to participate in 1-hour group-based sessions (medical assessment).
- Able to leave home on their own at least twice per week (e.g. for doctors visit, shopping)
- Patient's written informed consent has been obtained

4.2 Exclusion Criteria

- Medical conditions limiting participation (e.g. terminal illness, heart failure NYHA III-IV, unstable angina pectoris, uncontrolled seizure disorder, decompensated lung disease with less than ordinary activity leading to dyspnoea, cancer in advanced stage, chemo- or radiotherapy ongoing or during last three months, amputation of one or both legs).
- Complete dependence on rollator (wheeled walker).
- Visual, hearing or language problems affecting understanding of trial documents and procedures.
- Major cognitive impairment (MoCA score 18 or less).
- High risk for individual support during group session.
- Urge incontinence affecting group sessions.
- Severe personality disorder, psychiatric disease, alcohol use disorder or other substance use disorders affecting participation of group classes and regular practicing.
- Life expectancy of less than 1 year.
- Permanent confinement to bed to be expected in less than a year.
- Participating in regular Tai Chi or EYT exercises within last 6 months.
- Participating in vigorous sports for exercise within last month (e.g. training in fitness club or other sport activity inducing sweating and breathlessness or skiing and mountaineering in high mountains).
- Patient without legal capacity who is unable to understand the nature, significance and consequences of the study.

- Participation in a clinical trial within the 3 months before the start of this study (screening) or simultaneous participation in another study which could interfere with this study.

5 Recruitment and Enrolment of Subjects in the Study, Randomisation

5.1 Patient recruitment

A recruitment strategy will be set up to enrol the patients, but particularly to also invite economically disadvantaged patients, patients living a reclusive and less active life: The recruitment strategy includes:

- announcement through local media (magazine articles; radio interviews, display)
- announcement through a website: https://www.uniklinik-freiburg.de/entaier-studie.html
- flyers and posters
- oral presentations in nursing/retirement homes, meetings of senior citizens;
- personal contact (in person or via telephone) of the following persons and institutions, to inform them about the trial, provide trial brochures and flyers so they can inform patients about the trial:
    - local resident primary care physicians, orthopaedic doctors, neurologists, internists, dentists,
    - geriatric wards and ambulances, outpatient and inpatient trauma surgeries (to address patients after falls),
    - local nursing and retirement homes,
    - outpatient nursing services, household help,
    - other organisations providing help at home or working in the community (e.g. neighbourhood assistance, “meals on wheels”, social workers, priests),
    - social welfare office,
    - Senior Citizens' Office (*Seniorenbüro*), Senior Council (*Seniorenrat*),
    - organisations representing elder people (e.g. *Caritasverband, Diakonie, BAGSO, Paritätischer Wohlfahrtsverband, Landesseniorenvertretung, Kuratorium Deutsche Altenhilfe, Gesundheit aktiv*),
    - health insurances and their local representatives, therapists, Tai Chi and EYT organizations.

These will inform patients about the trial (oral and handing out flyers and address of website) and encourage them to get into contact with the local study sites.

5.2 Patient enrolment

Patients being informed about the trial and being interested will contact the local study sites or study teams.

Pre-screening

A pre-screening will then be done by telephone or in person. Information about the trial is given; inclusion and exclusion criteria as well as issues of compliance (regarding randomised

treatment allocation, documentation, EYT or Tai Chi group sessions, training at home, visits, follow-up) are discussed.

Waiting time

Patients that appear to be eligible for the trial and are interested in participation will enter a waiting time, until 15 or 30 patients could be enrolled.

Final Screening, Baseline, Enrolment

After the waiting time, patients that appear to be eligible, are still interested and have free time to join the EYT and Tai Chi group session, are invited for detailed information, final screening (see section 4) and baseline assessment (see section 6.3).

Final screening will be done by the investigator in person. Patients will receive detailed information about the trial. The patient will be asked for his/her written consent. It is imperative that written consent is obtained prior to any trial-specific procedures, including BBS, MoCA, which are needed to check the eligibility. Patients are asked to provide a list of all medications from their primary care doctor and may also be asked to provide medical reports (e.g. from last hospital stay or consultant) to check eligibility. Patients fulfilling inclusion criteria and giving informed consent will then have baseline assessment (see section 6.3).

The investigator will then record the details of these trial patients on the following trial-specific lists:

- Subject Screening log: for the documentation of the trial patients who were checked for eligibility. The following will be entered: the identification code*, the dates of written consent, and screening, as well as details of whether the patient was enrolled in the trial and, if not, the reason for not enrolling the patient.

- Subject identification log: A confidential log of the names of all trial patients with the identification code* assigned to each patient at the time of enrolment in the clinical trial. With this list, the identity of each patient can be revealed. The list must be kept confidential and must not leave the institution. It must remain at the study site and must not be copied or otherwise passed on! CRAs, auditors and representatives of authorities must be allowed to inspect the list on request.

* Patient identification code: A unique trial-specific identification number will be assigned to the patients. The first 2 digits correspond to the number of the study site, the next 3 digits stand for the consecutively screened patients at the particular study site, for example: 01001 (study site 1, patient 1), so that each patient is numbered uniquely across the entire database.

Start of treatment and observation period

After baseline assessment, patients are randomly allocated to the three groups. EYT and Tai Chi sessions (see section 8) have to start within 4 weeks after baseline assessment. The study books have to be handed over to the patients before the first EYT and Tai Chi session starts,

preferably at the same place and the same day but not more than 7 days before. The content of the study books has to be explained, as well as the visits, the fall prevention brochure and particularly also the documentation of falls. The group receiving standard care alone will be invited to the study site and receive the study book and concerning explanations at a comparable time (not more than 10 days apart). Patients will be recommended to visit their primary care physician to discuss fall prevention measures. Observation period and documentation starts with handing out this study book.

The study book contains: general instructions regarding the study book, contact information, a calendar, the fall diary and instructions how to use it, the brochure on fall prevention, a page for writing down their personal goals, pre-franked envelopes to send back the fall diary every month. The study book for patients allocated to the EYT and Tai Chi groups also contains: training diary and instructions how to use it, treatment manuals and instructions on how to practice regularly and safely, a DVD and an internet-link for a video for practicing.

5.3 Randomisation – general and methodological considerations

After baseline assessment (see sections 5.2 and 6.3) batches of ideally 15 patients should be randomised to the 3 research arms: EYT or Tai Chi or standard care alone (control). In exceptional cases – e.g. when recruitment is much slower or faster than expected - 12 or 18 patients can be randomised at once, respectively.

If possible, two batches of 15 (or 12 or 18) patients should be enrolled and randomised to 2 x 3 groups. If two batches with 15 (12, 18) patients are randomised concurrently (or within a few days), it is recommended to subdivide the batches depending on their risk of falling (BBS), to randomise one batch of 15 (12, 18) patients with lower BBS and one batch of 15 (12, 18) patients with higher BBS.

The randomisation code will be generated by the CTU using the following procedure to ensure that treatment assignment is unbiased and concealed from patients and investigator staff. For each batch size (12, 15 and 18) a separate randomisation fax form and randomisation lists will be prepared. Randomisation will be stratified by clinical site and performed in blocks of length 12, 15 or 18 in a ratio of 1:1:1. The randomization code will be produced by validated programs based on the Statistical Analysis System (SAS).

Central randomisation by fax

The patient identification codes of the batch of patients assigned for the trial (see section 5.3) will be entered on the randomisation fax form. There will be randomisation forms and lists for 12, 15 and 18 patients respectively and it is important to check if the correct form was used. For each patient the questions on informed consent, and inclusion/exclusion criteria on the form will be answered and the BBS Score will be entered. The fully completed form will then be faxed to the central randomisation office (Clinical Trials Unit, CTU) for randomisation:

Clinical Trials Unit

Medical Center – University of Freiburg

Fax: +49 761 270-74 390

The CTU will review the patient's details on the randomisation fax and randomise the patients. The result of the randomisation will be send to the investigator.

The treatment can be initiated for each patient group according to the randomised treatment arm. If the details on the randomisation fax appear incomplete or implausible, the CTU will send the investigator a query fax for clarification.

6 Study Procedures

6.1 Visit schedule and assessments - Flowchart

Table 1 Visit schedule and assessments – Flowchart of the Trial

VISITS	Pre-Treatment (Screening, Waiting time, Baseline/Visit 1 Randomisation)				Treatment						Post-Treatment Follow-up	
						Phone Visit 2-3 (b)	Visit 4 (c)	Phone Visit 5-6	Visit 7 (c)	Phone Visit 8-12 (b)	Last Visit, EOT (d)
TIME			Week -4 to Day -1		Day 0	Month 1 & 2	Month 3	Month 4 & 5	Month 6	Month 7-11	Month 12
Pre-Screening	X										
Waiting time		X									
Randomization (a)				X							
Informed consent			X								
In-, exclusion criteria			X								
Demographic Data			X								
Handing out Study Book					X						
Fall prevention information					X						
Recommendation to visit primary care doctor					X						
Start treatment or control per patient (e)					X						
Review fall diary						X	X	X	X	X	X
BBS			X				X		X		X
MoCA			X				X		X		X
IADL			X				X		X		X

VISITS	Pre-Treatment (Screening, Waiting time, Baseline/Visit 1 Randomisation)				Treatment					Post-Treatment Follow-up	
						Phone Visit 2-3 (b)	Visit 4 (c)	Phone Visit 5-6	Visit 7 (c)	Phone Visit 8-12 (b)	Last Visit, EOT (d)
Medications			X				X		X		X
Patient reported: Baseline Questions			X								
Patient reported: Short FES-I			X				X		X		X
Patient reported: GDS-15			X				X		X		X
Patient reported: SF-12			X				X		X		X
Patient reported: ICPH (only EYT and Tai Chi group)							X		X		
Patient reported: FIMA			X				X		X		X
Safety; complications						X	X	X	X	X	X
6 month questionnaire									X		

(a) The randomisation fax should be prepared as close as possible to the treatment.

(b) Phone visits can be conducted up to +10 days

(c) Visit 4 and 7 (month 3 and 6) can be conducted within ± 2 weeks

(d) Last visit (month 12) can be conducted ± 4 weeks

(e) The treatment should start at the same day or one day after but not more than seven days after handing out the study book

6.2 Study procedures at Study Sites

See also (“Recruitment and Enrolment of Subjects in the Study, Randomisation”, section 5.)

The investigator will

- inform the subject about the project and ask for written consent (see section 16.1). The original consent form will be filed with the patient's source documents, the second form stays with the patient.
- ensure that only patients who meet all the inclusion and none of the exclusion criteria and which have free time to join the group sessions are randomised into the trial.
- a copy of the consent form needs to be filed in the investigator site file.
- organize or supervise the organization of appropriate rooms for group session (together with therapists and teachers).
- inform/supervise informing the EYT therapists and Tai Chi teachers about 1) recruitment progress, 2) expectable and 3) actual start of group sessions, to ensure timely start of the intervention.
- in case, therapists or teachers are unable or decline to conduct group sessions, supervise their timely replacement with qualified therapists or teachers and their instruction, supported by manuals provided by the Coordinating Investigator.
- supervise assessment of BBS, MoCA and filling in questionnaires (see section 2)
- ensure timely handing out of study book and start group sessions (at the most 4 weeks after baseline assessment)..
- in case that the patient withdraws his/her consent, the referring physician will complete a special form and inform the Clinical Trials Unit, Medical Center – University of Freiburg, via fax: +49 761 270-74 390. The following information is transmitted: date of withdrawal, information whether the patient has given consent that his data collected up to the date of withdrawal can be retained for further usage in the study. This document need to be stored into the investigator site file (after information via fax).
- Investigator or study personnel instruct the patients how to report falls in the diary.

6.3 Baseline Assessment:

Sociodemographic data, history, diagnoses, risk factors including:

- Age, gender, highest completed level of education and training, height, weight, BMI.
- Falls within last 6 months, with/without injuries), use of walking aid.
- Trouble vision, trouble hearing, vertigo, trouble walking.
- Diagnosis, (cardiovascular, orthostatic dysregulation, pulmonal, diabetes, other internistic, osteoporosis, mental condition, sleep disorder, history of stroke,

Parkinson disease, multiple sclerosis, polyneuropathy, other neurological, chronic arthritis of knee or hip or feet, other musculoskeletal).

- Sport/physical activity
- Social activity (living alone, with family, friends; socializing/meeting with friends, relatives, neighbours; social support through family, friends, neighbours.

The document asking for diagnoses should be signed by the investigator to make sure that patients understood and filled it out correctly

BBS is assessed by a study personnel, as well as MoCA and IADL, supported by written instructions.

The questionnaires *Short Falls Efficacy Scale-International, GDS-15, SF-12, ICPH, FIMA* are filled in by the patients. Study personnel will support patients to fill in questionnaires, particularly the FIMA, and check them for completeness. Also family members/next of kin or health care professionals can support patients to fill in the questionnaires (see do's and don'ts at table).

The questionnaires should be completed in the same environment, quiet and respectful of the subject's privacy and prior to other investigations. Patient reported questionnaires are to be filled out by the patient him/herself. It is important that the investigator or other study personnel do not influence the patient in any way during the completion of the questionnaire (see do's and don'ts at table).

The following checklist contains some "do's" and "don'ts" when administering the questionnaires (except baseline questions).

DOs	DON'Ts
**Do** have the subject complete the questionnaire and make sure questionnaire are filled in completely	**Do not** discuss the subject's health, health data, or emotions with them before they fill out the questionnaire
**Do** be warm, friendly and helpful	**Do not** force or command subjects to fill out the questionnaire
**Do** request and encourage subjects to fill out the questionnaire	**Do not** accept an incomplete questionnaire without first encouraging the subject to fill out unanswered questions
**Do** tell the subject to answer a question based on what they think the question means	**Do not** interpret or explain a question
**Do** have the subjects fill out the	**Do not** allow the wife/husband or

DOs	DON'Ts
questionnaire by themselves	family members to help the subject complete the questionnaire
**Do** encourage subjects to complete all questions	**Do not** minimise the importance of the questionnaire
**Do** inform subjects that they will be asked to fill out another questionnaire at a later date (if applicable)	**Do not** change any question since this will affect the scoring

The questionnaire MoCA is filled in by the investigator/physician or qualified study personnel on the basis of an interview with the patient or, if needed, by a family member/next of kin.

Data obtained from assessments done by the investigator or study nurse, as well as data obtained from patient's fall diary and training dairy will be filed with the patient's source documents and documented into the eCRF by designated personnel of the study site (e.g. investigator or study nurse). Patient-reported questionnaires will be stored at the patient's source documents and a duplicate sent to the Clinical Trials Unit in Freiburg.

6.4 Treatment Phase and Follow-up Assessments

Patients will record falls in a diary. The diaries will be sent to the study site at the end of each month (or up to 7 days later) until month 12. Patients will be interviewed at the end of each month (or up to 10 days later) to rectify missing data and to ascertain details of falls (see section 2.2), injuries and severity (see section 2.3), and complications (see section 10) by study personnel. If the diary is not send, investigator/physician or study personal will contact the patient.

Patients will visit the study site in person for follow-up evaluations at completion of month 3, 6, 12 (+/- 2 or 4 weeks, see Flowchart, section 6.1) for visit 4, 7, 12. The investigator will send a reminder to the patient. If a patient is not able to come for a study visit, every possible effort is made to collect follow-up evaluations. If feasible, follow-up evaluation can also be conducted at patients home. BBS is assessed and questionnaires are filled in by the trial investigator and/or by the study nurse or the patient (see section 2).

Outcomes measures should be conducted, as far as possible, at a consistent time of the day and consistent to medication cycles, particularly in patients with conditions like Parkinson and Multiple sclerosis, who use medications directly influencing mobility and balance.

Attempts should be made to collect all questionnaires for all patients. Particularly the "standard care alone" patients should get special attention by the investigators to reduce loss of follow-up

in this group. However, if the patient declines to complete the questionnaires, this should be documented in the CRF.

Patients should be given sufficient space and time to complete the study questionnaires and all administered questionnaires should be reviewed for completeness. If missing responses are noted, patients should be encouraged to complete any missing responses.

Investigators must not encourage the patient to change responses reported in questionnaires.

Data obtained from assessments done by the investigator or study nurse, as well as data obtained from patient's fall diary and training dairy will be filed with the patient's source documents and documented into the eCRF by designated personnel of the study site (e.g. investigator or study nurse). Patient-reported questionnaires will be stored at the patient's source documents and a duplicate sent to the Clinical Trials Unit in Freiburg.

Safety assessments

Safety will be monitored by collecting information on complications at every visit. For details on complications and their collection see section 10.1.

Lost to follow-up

Patients lost to follow-up should be recorded as such in the CRF. For patients who are lost to follow-up, the investigator should show "due diligence" by documenting in the source documents steps taken to contact the patient, e.g., dates of telephone calls, registered letters, etc.

EYT therapists, Tai Chi teachers

EYT therapists, Tai Chi teachers will document at each group session the patients that have participated. They will document monthly whether they deviated from the manual and the reasons for deviation. They will sign the participation of each session in patient's diary. They will instruct the patients how to report the training in their training diary.

7 Premature Termination or Suspension of a Study

7.1 Premature termination of one of the treatment arms or the entire trial

The coordinating investigator is under obligation to monitor the progress of the clinical trial with regard to safety-relevant developments and, if necessary, initiate the premature termination of a treatment arm or the entire clinical trial. The coordinating investigator will be supported in this responsibility by the Data Monitoring Committee (DMC).

A treatment arm or the entire clinical trial must be terminated prematurely if:

- the benefit-to-risk ratio for the patients changes markedly,
- the coordinating investigator (German LKP) or the DMC considers that the termination of the trial is necessary,
- indications arise that the trial patients' safety is no longer guaranteed,
- an insufficient recruitment rate makes a successful conclusion of the clinical trial unrealisable/no longer feasible.

If the clinical trial is prematurely terminated, the IEC(s) (where required by the applicable regulatory requirements) will be informed (this is usually done by the coordinating investigator).

7.2 Premature termination of the trial at one of the study sites

The investigator and the coordinating investigator have the right to terminate the trial at one of the sites.

The clinical trial can be terminated prematurely at the study site by the investigator if, for instance, unforeseeable circumstances have arisen at the study site which preclude the continuation of the clinical trial, the investigator considers that the resources for continuation are no longer available, the investigator considers that the continuation of the trial is no longer ethically or medically justifiable.

The coordinating investigator can initiate the exclusion of a study site from further participation if, for instance, patient recruitment is inadequate, serious problems arise with regard to the quality of the collected data which cannot be resolved.

Premature termination at one of the study sites does not automatically mean a termination of the trial for already enrolled trial patients. A separate decision on further treatment must be made for each patient, depending on the overall situation. Follow-up of already enrolled trial patients must be ensured. The documentation of already enrolled trial patients will be reviewed for completeness and plausibility. Queries may be raised for further clarification before the study site is closed. These queries must be answered properly by the site. The CA(s) and IEC(s) must be duly notified of the study site´s closure, including reasons, within the specified period. The

study site concerned will be closed in stages by the CRA when a decision has been made on the further treatment of the patients concerned.

7.3 Premature discontinuation of trial participation for individual patients

The trial patient can withdraw consent for trial participation e.g. consent for the follow-up visits and documentation at any time, without having to give reasons.

The responsible investigator may only withdraw a patient from participation in the trial for the following reasons:

- Extreme circumstances arise which make any trial-relevant follow-up impossible

The documentation should be completed as far as possible under these circumstances, e.g. a final examination and documentation according to the protocol (if possible), a documentation of the premature trial termination on the CRF and in the medical record, giving reasons, should be ensured.

7.4 Premature discontinuation of trial treatment for individual patients

The trial patient can have his/her trial treatment terminated prematurely at any time, without having to give reasons.

The investigator responsible for the trial has the right to terminate the treatment of a patient according to the following conditions:

- Adverse events (including intercurrent illnesses) which preclude further treatment with EYT or Tai Chi or make further participation in the clinical trial inadvisable because the informational value of the trial results is impaired.
- Premature termination of the trial treatment is considered to be medically indicated, e.g. because it is subsequently found that inclusion/exclusion criteria were severely violated.
- Continuation of the trial treatment is unacceptable when the risks outweigh the benefits or in case of a significant deterioration of a health condition or a new diagnosis during month 1-6, affecting safety of the patient and participation in group classes.
- Logistical reasons (patient moves to another location).

In the case the trial treatment of a patient has been stopped prematurely, further follow-up visits and the assessment of the trial endpoints are essential to enable an analysis of the full analysis set according to the intention-to-treat principle. Further visits, follow-up and documentation should always be striven for/ensured in this case. This includes the follow-up of primary and secondary endpoints, as well as complications. The time of termination, and the reason for termination should be documented in the CRF.

8 Investigational Interventions

After randomised treatment allocation all study patients will be recommended standard care. Patients allocated to EYT and Tai Chi will start with group exercises.

8.1 Intervention description and application

Brief Name: Eurythmy Therapy (EYT), Tai Chi/Taiji, standard care.

What: EYT [12, 13] and Tai Chi [7-10] are mind-body oriented exercise therapies, training body movements as well as concentrative, sensory and mindfulness capacities. They pursue comparable movements, smoothly flowing, creating sensation and awareness of limb movement and balance (see 2.2). Tai Chi is instructed and guided by a trained teacher giving oral instructions and helping with conducting exercises. EYT is instructed and guided by a therapist, demonstrating and helping with exercises; EYT exercises are accompanied by music, phonems, poems or rhythms. Patients additionally receive oral and written and audio-visual instructions (see study book) on how to practice daily at home between group sessions. Patients will keep a training diary. Each subsequent group session will start with a short appreciating and constructive discussion on the experience with daily exercises, and what was regarded as supportive.

A core set of exercises are described in a manual and in a video handed out as a DVD to the patients to support them for exercises in between the group sessions. These exercises are based on prior studies investigating fall prevention or on recommendations by experts applying them in the target population, and adapted to the balance and motility limitations expected in the study patients. They have been peer reviewed by participating Tai Chi teachers, EYT therapists and external experts. Beyond, therapists and teachers are allowed to individually tailor and modify exercises to the capabilities of the patients.

“Standard care” means: patients will receive a brochure with detailed written evidence-based recommendations to reduce risk of falling, clearly illustrated and prepared to specifically address the age population *(“Gleichgewicht & Kraft – Einführung in die Sturzprävention”* https://www.bzga.de/infomaterialien/gesundheit-aelterer-menschen/gleichgewicht-und-kraft-einfuehrung-in-die-sturzpraevention/)). Patients will also be recommended to visit their primary care physician if they have not done so within the last three months, to discuss fall-prevention measures. Besides the guidance of the brochure, primary care physicians have recent evidence-based guidelines on geriatric assessments including fall prevention measures [60], on managing multi-morbidity [61], and polypharmacy [62]. Current guidelines for fall prevention as well as the fall prevention brochure refer to active lifestyle; to specific exercises to improve balance, strength, flexibility, endurance; to medication modification; to safe home environment; to vitamin D supplementation; to safe footwear; to assistive devices; to improving medical conditions, to treat vision impairment, to manage postural hypotension, heart rate and rhythm abnormalities [22, 23]; the fall prevention brochure also provides addresses. Fall prevention

measures will be organized by the primary care doctors (e.g. prescriptions or referrals) or can be conducted by the patients themselves, using the recommendations of the brochure.

Therapists and Tai Chi teacher will receive a refresher training in appreciating, encouraging, non-judgmental communication (see section 9), before commencement of the trial. They also receive written proposals regarding inner attitude and regarding communication in the exercise groups and in challenging situations. In a follow-up internet-based meeting, they have the opportunity to ask questions and get additional advice. They will receive recommendations to support motivation and adherence of patients. This shall help them to encourage patients to participate in group sessions and to regularly exercise at home and to manage challenges of the group situation.

Study personnel will also receive a refresher training in appreciating, encouraging, non-judgmental communication (see section 9), before commencement of the trial. They also receive written proposals regarding communication with the patients, in a long version and as a short memo card. This is meant to help them to support patients participating in the trial and to appreciate their commitment and effort and to address their need for a calm, encouraging, non-judgemental communication.

Who provides: EYT will be provided by a certified EYT therapist (at least 5 1/2 years of training according a standardized curriculum, acknowledged by the “Eurythmy Therapy Association” of Germany) and with experience in treating people comparable to the trial population.

Tai Chi will be provided by a teacher, with at least 500 hours training according to a standardized curriculum certified by the *“Bundesvereinigung Taijiquan und Qigong-Netzwerk”* or by the *“Deutscher Dachverband Tai Chi und Qigong”* or to an equivalent curriculum or an equivalent of 500 hours of own teaching Tai Chi (e.g. Tai Chi-teacher which have trained before commencement of these curricula or in other countries), and with working experience in instructing people comparable to the trial population.

Therapists and teachers are qualified and experienced in instructing and guiding the exercises. Before commencement of the trial, the therapists and teachers are invited to practice together. If unable to attend or if recruited at a later time, they will be informed by a manual, by investigator and study personnel of the study sites and by other participating therapists and teachers. They can also communicate with each other via Email and in telephone/web conferences before and during the trial.

Therapists and teachers receive a folder containing general instructions, the manual, participation lists, a documentation sheet, the motivational concept and the communication concept and proposal.

Standard care is provided and organized by the patients’ primary care doctor or consultant according to the patient’s own discretion. The brochure “*Gleichgewicht & Kraft - Einführung in die Sturzprävention*” (https://www.bzga.de/infomaterialien/gesundheit-aelterer-menschen/gleichgewicht-und-kraft-einfuehrung-in-die-sturzpraevention/) providing detailed evidence-based recommendations to reduce risk of falling and enable safer mobility is provided

by the study sites. It also enables the patient for self-help. Neither therapists nor teacher nor study personal is meant to comment, recommend or dissuade on any standard care measures or any other fall prevention measures.

How: EYT and Tai Chi will be provided in one-hour group sessions, á 5 (± 1) patients. These group sessions start with an appreciating discussion about practicing (see section 9), followed by practicing the exercises and – if appropriate – ending with a resting time (approximately 15 minutes). Patients are instructed to practice at home at a minimum of 20 minutes on 3 non-class days.

Where: EYT and Tai Chi will be provided in appropriate rooms: They should be easily accessible with public transportation, if possible, located nearby the patients (e.g. in retirement homes, meeting places of senior citizens, churches, at the study sites, in doctor's facilities or in rooms provided by EYT therapists or Tai Chi teachers). The room should be accessible for those with disabilities, should support the patients to feel comfortable, to exercise freely (size 50-70 m^2, minimum 36 m^2), friendly bright, quiet, without distraction, properly ventilated and heatable, with no cold stone floor. One chair per patient (stable and safe) has to be provided. Restrooms should be easy accessible, on the same flour.

When: EYT and Tai Chi will be provided first twice/week (week 1-12) and then once/week (week 13-24). The intensity is higher in the first 12 weeks (twice per week), taking into account the learning curve, and reduced to once per week during the second 12 weeks when individually practicing has become more routine. Participants are instructed to practice exercises at home.

Tailoring: Tai Chi and EYT exercises follow the manuals but are individually tailored to the capabilities of the participants. When exercises beyond the manual are used, this will be documented by the EYT therapists or Tai Chi teacher, together with the reason for using them. In case of significant deterioration of health condition or a new diagnosis during month 1-6, affecting safety of the patient and participation in group classes, the EYT therapist or Tai Chi teacher should contact the investigator whether group sessions should be interrupted.

How well: Patients will give feedback at the beginning of each session regarding their adherence to and their experience with practicing at home. Patients will keep training diaries. Patients will visit group sessions. Deviations from the manual will be documented by therapists and teachers.

8.2 Additional interventions

Following additional treatments should not be used during the trial:

- EYT in the Tai Chi group and standard care alone group
- Tai Chi, Qigong, Yoga in the EYT group and standard care alone group

All other treatments are allowed. As Tai Chi and EYT are investigated as an add-on to standard care, no interventions are prohibited, except the study intervention (as well as Qigong and

Yoga) in the comparison groups. On the contrary, a brochure providing detailed evidence-based recommendations to reduce risk of falling and enable safer mobility is provided to all patients

9 Support of motivation and adherence

Compliance will be assessed by the Investigator and/or study personnel at each patient visit.

A patient diary regarding training will be provided for each patient at visit one and need to be filled in during the entire trial.

Adherence and motivation will be increased by

- appreciating group sessions,
- discussing supporting and avoiding discouraging elements,
- contacting the patients via phone to remind them on their appointment during the first treatment weeks (subsequently, this reminder can be continued depending on the needs of the patients),
- oral, audio-visual and written instructions on how to practice daily at home between group sessions,
- keeping a training diary,
- writing down own goals in the study book,
- starting each group session with an appreciating and constructive discussion on the experience with daily exercises,
- training of investigators, study personnel, therapists, teacher in communication techniques that take regard of motivating factors especially for the older patients (e.g. appreciating, encouraging, non-judgmental, unagitated, giving time, calm),
- a supportive community, giving sufficient time, achievable, individually adapted exercises [26-28].
- Special attention and appreciation is given to all patients, including the patients allocated to “standard care alone”, to address their needs and increase their feeling of being seen and appreciated, and their adherence with study procedures.

A summary of research on motivation of elderly to exercise as well as of personal recommendations by experts in the field of motivating elderly (geriatric care, pedagogy for social gerontology, trainers of fall prevention exercise classes, communication) will be handed out to EYT therapists and Tai Chi teacher.

In summary it recommends to adapt the approach in communication and motivation to the special needs and habits of elderly people. The patients should be informed about the positive effects of exercise. Personal health believe models should be addressed. Self-efficacy should be shown using easy exercises at the beginning, where the patients can already experience success. Exercises for training at home should firstly be simple, and less demanding. Short-term and long-term successes should be named.

The group sessions should create an open and safe atmosphere, where every patient is seen and valued in his actual situation. New exercises can be embarrassing for the patients as they feel shame not doing it right while others are watching. This has to be considered carefully and

appraisal within the group has to be led by the group leader. Patients should be asked for personal goals which they might achieve with the exercise – like going to a special place or helping their grand-children. Individual patient concerns have to be addressed like the fear of falling and injuries. Patients should be individually interviewed, whether a current disease limits exercise and whether this can be treated or if the exercises should be adapted to this condition. Patients should participate in designing and organizing the group session. The patients should have the opportunity to socialize before or after the group session.

Investigators, study nurses, assistants, therapists and Tai Chi teacher will receive a refresher training in appreciating, encouraging, non-judgmental communication, before commencement of the trial. In a follow-up internet-based meeting, they have the opportunity to ask questions and get additional advice. They are handed out recommendations for appreciating, encouraging, non-judgmental communication.

Patients will be compensated for travel costs to exercise and to visits: 90,- € for first 6 months and 20 € for first screening and follow-up. In case the study centre decides to conduct the follow-up visits at the home of the patient, the compensation (20,- € per visit) will then be used for this purpose.

If participation of patients in group sessions depends on reimbursement of travel costs (social welfare, *“Grundsicherung”, “Hartz 4”),* up to 15 % of patients regularly visiting EYT- or Tai Chi-group sessions can receive further reimbursement of public transportation costs to visit group sessions (for further details see study site contracts).

10 Safety monitoring and reporting

10.1 Complications

10.1.1 Definition of complications

Only complications that could arise from the study specific intervention (EYT or Tai Chi or other exercises in the standard care group) will be collected, documented and – in case of serious complications – reported to the Data Monitoring Committee, DMC (details see chapter 10.3 and DMC Charter). A complication in the ENTAiER study is defined as any untoward medical occurrence in a trial patient such as any unfavourable and unintended sign, symptom, or disease that is possibly related to the study intervention. As the (non-pharmacological) intervention (EYT and Tai Chi) is training of musculoskeletal, sensory, concentrative elements, complications can be:

- Injuries caused by falls that happened during exercises in groups or at home.
- Musculoskeletal pain – except aching muscle - (e.g. foot, knee, low back pain, arthritis, joint effusion, reduced joint function), neuralgia, cardiovascular disorders (e.g. dizziness or faintness, symptoms of hypotension or hypertension, syncope, chest pain, cardiac arrhythmia), vertigo, dizziness, fatigue, syncope, dyspnoea, bronchospasm, dyspnoea, neurological cognitive disturbance, confusion, mood alteration, or others.

Not regarded as complications are

- conditions that were already present at the time of informed consent
  aching muscle (‘Muskelkater’), which is to be expected.

The expression “related” means, that there is evidence or argument to suggest a reasonable causal relationship between the event and the administration of the intervention, e.g. close temporal connection, exclusion of other causes.

The assessment “not related” is appropriate, if the complication is clearly or most likely explained by other causes even if a potential relationship between treatment and the complication cannot be completely excluded.

Falls and injurious falls will be documented as primary outcomes. In case they happen during exercises in groups or at home, this will be noted. They will also be reported to the study center by therapists or teachers in case that they happen during exercises. Injuries caused by falls that happened during exercises in groups or at home will be documented as complications and indicated as caused by a fall. All falls that happen during exercises in groups or at home will be reported to the DMC with the regular report on complications.

Further information on safety will be referred from the FIMA, assessing health care utilization like physician contacts and hospitalizations at month 3, 6 and 12.

10.1.2 Documentation of complications

All such complications as defined in 10.1.1 reported by the patient or observed by the investigator, teacher or therapists will be continuously documented in the designated case report form (Complications form) during the clinical trial/at each follow-up visit.

Patients will be asked using open questions regarding their condition and any untoward medical occurrences during the last four weeks or any unplanned visits to a doctor, ambulance or hospital. In case of an occurrence, causal relationship with the study intervention EYT or Tai Chi, or, for instance in case of patients allocated to standard care alone, with physical training will be assessed.

In case of a potential causal relation to the intervention, the complication has to be documented in the CRF starting from the first administration and until at least 7 days after the last administration of the intervention.

Following information will be documented:

- Characterization of the event
- Onset date, if available
- Severity: see “assessment of severity” (see Section 10.1.3))
- Serious or non-serious
- Action taken with Intervention
- Outcome

10.1.3 Assessment of Severity

The intensity of the complication will be rated as mild, moderate, or severe using the following criteria:

- ***Mild***: These events require minimal or no treatment and do not interfere with the subject’s daily activities.
- ***Moderate***: These events result in a low level of inconvenience or concern with the therapeutic measures. Moderate events may cause some interference with functioning.
- ***Severe***: These events interrupt a subject’s usual daily activity and may require systemic drug therapy or other treatment. Severe events are usually incapacitating.

Changes in the severity of a complication should be documented to allow an assessment of the duration of the event at each level of intensity to be performed.

10.2 Serious Complications

10.2.1 Definition and documentation of serious complications

In compliance with ICH-GCP (guideline E6 [R2]) section 4.11 and 5.17 we have taken the decision that the documentation and expedited reporting should be limited to events showing a causal relationship to the study procedure. For this reason, the investigator is obliged to document only the following events:

- Death of the patient,
- Life-threatening situation (patient is at immediate risk of death),
- Persistent or significant disability/incapacity.

Clarification of serious complications:

NOTE: The term “life-threatening” in the definition of “serious” refers to an event/reaction in which the patient was at risk of death at the time of the event/reaction; it does not refer to an event/ reaction which hypothetically might have caused death if it were more severe,

Please note that “death” is usually an outcome of a complication and not a serious complication per se. Only in cases where the clinical circumstances before the death are unknown (i.e. patient died without a determinable cause of death), then the diagnosis “death” itself should be reported as a serious complication. In case of fatal outcome of an already-registered serious complication, a follow-up notification must be done.

10.2.2 Documentation of serious complications

All serious complications that occur starting from the first intervention and until at least 7 days after the last intervention will be documented in the eCRF.

11 Data Monitoring Committee

An independent data monitoring committee (DMC) will be established with the members listed in the section "Responsibilities" of this trial protocol. The function of the DMC is to monitor the course of the study and if necessary to give a recommendation to the Coordinating Investigator and sponsor of the trial for discontinuation, modification or continuation of the study.

The underlying principles for the DMC are ethical and safety aspects for the patients. It is the task of the DMC to examine whether the continuation of this trial is still ethically justifiable, whether the safety of patients is ensured, and whether trial progress is acceptable. For this purpose, the DMC has to be informed regularly about patient recruitment, compliance, and safety. The DMC will also receive the corresponding reports. The composition and responsibilities of the DMC, the structure and procedures of its meetings, and its relationship to other key study team members will be laid down in a separate DMC charter.

12 Data Collection, Handling and Management

12.1 Data confidentiality

Information about trial patients will be kept confidential and managed under the applicable laws and regulations. Those regulations require a signed patient authorization informing the patient of the following:

- what protected health information (PHI) will be collected from patients in this trial;
- who will have access to that information and why;
- who will use or disclose that information;
- the rights of a research patient to revoke their authorization for use of their PHI.

All subject data will be captured in pseudonymised form.

The electronic data capture (EDC) system utilized in this trial uses built-in security features to encrypt all data for transmission in both directions, preventing unauthorised access to confidential participant information. Access to the system will be controlled by individually assigned user identification codes and passwords, made available only to authorised personnel who have completed prerequisite training.

12.2 Documentation of trial data

Documentation in electronic case report form (eCRF)

An electronic data capture (EDC) system will be used in this trial. The investigator will document all trial data in a trial-specific electronic case report form (eCRF), as timely as possible. Data entry and data corrections on e-forms are automatically tracked in the audit trail created by the EDC system.

Data corrections in the eCRF due to queries are performed by the responsible investigator, or designated person, as timely as possible.

12.3 Data management

The data management will be performed with REDCap™ Version 8.6.5, a fully web based remote data entry system based on web forms, which is developed and maintained by the REDCap Consortium (redcap@vanderbilt.edu).

Details on data management (procedures, responsibilities, etc.) will be described in a data management manual prior to the trial. The Data Management Manual is a working document

which will be continuously updated and maintained during the trial, i.e. the performance of data management and any deviations from the first version of the data management manual will be documented therein as well. The technical specifications of the database will be described in a data description plan (in short “DDP”). Before any data entry is performed, the trial database and edit checks will be tested. Data entry personnel will not be given access to the trial until they have been trained. An audit trail provides a data history of which data were entered, changed or deleted, by whom and when.

Data will be regularly reviewed for completeness, consistency and plausibility. The checks to be programmed will be specified beforehand in a data validation plan (integrated in the DDP stated above). After running the check programs, the resulting queries will be sent to the investigator for correction or verification of the documented data. Data corrections will be entered directly into REDCap by the responsible investigator, or a designated person.

12.4 Data coding

Complications will be coded using Medical dictionary for regulatory activities (MedDRA) terminology.

13 Quality Assurance System

The sponsor / coordinating investigator is responsible for implementing and maintaining quality assurance and quality control systems with written Standard Operation Procedures (SOP) to ensure that trials are conducted and data are generated, documented (recorded), and reported in compliance with the protocol, GCP, and the applicable regulatory requirement(s).

During the study, quality control and quality assurance will be ensured through monitoring, auditing and supervision.

13.1 Quality Control (Clinical Monitoring)

Monitoring is performed by the CRAs of the CTU, Medical Center - University of Freiburg. Risk-based monitoring will be done according to ICH-GCP (R2) and standard operating procedures (SOP) to verify that patients' rights and wellbeing are protected, reported trial data are accurate, complete and verifiable from source documents. This monitoring also verifies that the trial is conducted in compliance with the currently approved protocol/amendment, with ICH-GCP (R2) and with the applicable regulatory requirements to ensure safety and integrity of clinical trial data.

The investigator will grant the CRA access to the patients' personal medical records for the verification of the proper documentation of study data and agrees that the CRA will visit the study site before, during and after completion of the study for the fulfilment of his/her duty. The investigator must allow sufficient time for these visits, alternatively the CRA may be provided with other trained staff (e.g. so-called co-investigators) for assistance during the visits.

During the trial, the CRA will visit the study site regularly depending on the recruitment rate and quality of data. During these on-site visits, the CRA verifies that the trial is conducted according to the trial protocol, trial specific procedures, ICH-GCP (R2) and national/local regulatory requirements. The presence of signed informed consents, eligibility of patients, primary endpoint and documentation/reporting of safety data (e.g. (serious) complications) will be verified by the CRA.

A monitoring report will be written on each visit. This will document the progress of the study and give an account of all problems that occurred (e.g. refusal of inspection).

Between visits, the responsible CRA will maintain regular contact with the study sites. Further detailed information on monitoring will be recorded in a monitoring manual.

13.2 Source Data Verification (SDV)

Source data verification will be performed in order to verify the accuracy and completeness of the entries on the electronic case report form (eCRF) by comparing them with the source data, and to ensure and increase the quality of the data. The investigators will afford the CRA access to the medical records for the performance of SDV. Further detailed information on monitoring will be recorded in a monitoring manual.

Source data as defined by ICH-GCP (R2) include data such as hospital records, clinical and office charts, laboratory notes, memoranda, patients' diaries or evaluation checklists, pharmacy dispensing records, recorded data from automated instruments, copies or transcriptions certified after verification as being accurate copies, microfiches, photographic negatives, microfilm or magnetic media, x-rays, and records kept at the pharmacy, at the laboratories and at medico-technical departments involved in the clinical study.

13.3 Quality Assurance (Auditing)

According to the ICH-GCP (R2) guidelines, audits will be performed according to a quality assurance system. These may be conducted by the coordinating investigator.

During an audit, the planning, conduct and analysis of a study might be checked for compliance with the national laws and the requirements of the ICH-GCP (R2) guideline and will be stated by the conducting authority.

This might include checking the data management and organisation at the study site and inspecting institutions and source documents. The aim of quality assurance is to ensure that the results and conclusions described in the final report can be correctly deduced from the raw data.

All persons performing an audit undertake in writing to treat all patient-relevant data confidentially restrict the use of such data to the purposes agreed by the patient in writing.

Proposed audit dates, characteristics of the selected patients and further information will be transmitted to the investigator by the CRA in a timely manner.

14 Biostatistical Planning and Analysis

Before the start of the final analysis a detailed statistical analysis plan (SAP) will be prepared. This will be completed during the 'blind review' of the data, at the latest. This blind review, i.e. a checking and assessment of the data, will be performed after the end of the recruitment period and the planned follow-up period without looking at the randomised treatment for each patient. If the SAP contains any changes to the analyses outlined in the trial protocol, they will be marked as such, and reasons for amendments will be given.

All statistical programming for analysis will be performed with the Statistical Analysis System (SAS).

14.1 Study Design

Multi-centre, randomised controlled, open-label trial, stratified randomised by study site, with two active groups and one control parallel group.

14.2 Objectives and Endpoints

See Section 2.

14.3 Sample Size Calculation

Sample size calculation is based on assumptions on the proportion of patients with at least one fall during a 6-months period. The proportion of patients with at least one fall is assumed as 50% in the standard care group [50, 59]. A decrease of this proportion to 35% is considered as clinically relevant and as achievable by EYT as well as by Tai Chi [63]. The study is planned with 80% power to show a difference between treatments at two-sided level alpha of 5%, when proportions of fallers are 50% with standard care and 35% both with EYT and with Tai Chi. Based on the chi-square distribution, this requires 155 patients per group (calculated with NQuery Advisor 7.0). The sample size calculation was performed for the three-group comparison. To account for multiplicity issues in this three-group comparison, a closed test procedure at two-sided level alpha of 5% will be used, i.e. the global null hypothesis of no differences between any of the three intervention types will be tested first, and if it can be rejected, pairwise group comparisons will be conducted each at two-sided level alpha of 5%. The two-group comparison of EYT versus standard care has a power of 80%, when the proportion of fallers is reduced from 50% to 34.3%. The two-group comparison of EYT versus Tai Chi has a power of 80%, when the difference in the proportion of fallers is 15%. To account for a certain amount of non-compliance and incomplete observations, 550 patients will be randomised in a ratio of 1:1:1.

14.4 Definition of Populations Included In the Analyses

The trial will be analysed according to the ‘intention-to-treat principle’. That is, the primary analysis for comparison of the treatment groups will be based on the 'full analysis set' (FAS).

The FAS includes all randomised patients, and patients are analysed as belonging to their randomised arm, regardless of whether they declined therapy, or whether other protocol deviations are known.

Additionally, a comparison of the randomized treatment groups with regard to efficacy will be performed in the 'per-protocol set' (PP). This is a subset of the patients in the full analysis set and includes only patients for whom no major protocol violations are known. Comparisons of the treatments in the 'per protocol set' will be performed for the purpose of sensitivity analysis. Major protocol violations will be defined in the statistical analysis plan.

Safety analyses will be performed in the safety population (SAF). This is a subset of all randomized patients and includes patients who participated in at least one group session. Patients in the SAF will be analysed according to intervention received.

14.5 Methods of Analysis

14.5.1 Primary Endpoint

The primary efficacy analysis will be performed in the full analysis set (FAS). For the analysis of the primary endpoint fall (yes, no) within 6 months a time-to-event methodology will be applied using the time to the first fall to account for potentially incomplete observation times of the patients. This is considered to be more efficient than an analysis of the binary endpoint fall (yes, no), and therefore leading to a gain in power. The time to first fall will be calculated from date of handing out the study book to the first fall. For patients who do not fall within the 6 months observation period, the time to last contact will be used as censored observation. Death without previous fall will be regarded as competing event in the analysis. A Cox regression model will be used to test the efficacy of EYT versus Tai Chi versus standard care in reducing the risk of falling within 6 months after handing out the study book. The model will include intervention type, study site, BBS score, and age as independent variables. To account for multiplicity issues in this three-group comparison, a closed test procedure at two-sided level alpha of 5% will be used, i.e. the global null hypothesis of no differences between any of the three intervention types will be tested first, and if it can be rejected, pairwise group comparisons will be conducted each at two-sided level alpha of 5%. Hazard ratios (HR) between interventions will be calculated from the model with two-sided 95% CI. Cumulative incidences for both competing events will be graphically displayed. In addition to the overall comparison of the treatment arms, a comparison of treatments in key patient subgroups is of interest. The treatment effect will be estimated separately in patients defined by different diagnoses (present vs. absent) from regression models including the interaction between treatment and diagnosis. The diagnoses investigated include: cardiovascular, orthostatic dysregulation, pulmonal, diabetes, other internistic, osteoporosis, mental condition, sleep disorder, history of stroke, Parkinson disease, multiple sclerosis, polyneuropathy, other neurological, chronic arthritis of knee or hip or feet, other musculoskeletal.

Depending on the patients included in the trial, some of the diagnoses might not be analyzed, since there might be no patient with that diagnosis.

As a sensitivity analysis these analyses above will be repeated in the PP.

14.5.2 Secondary endpoints

For the analysis of the secondary endpoint total number of falls per patient within 6 months after handing out the study book, a negative binomial regression model will be used to test the efficacy of EYT versus Tai Chi versus standard care. Negative binomial is preferred over Poisson regression because of potential overdispersion. Fall rates for patients with follow-up times of less than 6 months will be adjusted by accounting for their exposure time. The model will include intervention type, study site, BBS score, and age as independent variables. Incidence rate ratios (IRR) between interventions will be calculated from the model with two-sided 95% CI. To take into account potentially present excess zeroes, a zero-inflated negative binomial model will be used as a sensitivity analysis

Other secondary endpoints will be analysed with appropriate regression models. Analyses will be repeated for the observation period of 12 months after date of handing out the study book. This also applies to the primary endpoint at 12 months.

As a sensitivity analysis these analyses above will be repeated in the PP.

Safety analyses will be performed in the SAF. Rates of complications will be calculated with two-sided 95% CI.

No formal interim analyses of the efficacy endpoints will be performed.

14.6 Economic Analysis

Utilization of health-care-cost will be calculated by applying standardized unit-cost allowing the evaluation of cost development for each group over time. Finally, at three points (baseline, month 6 and month 12), utilization of health-care-costs is recorded for every patient.

Costs of health-care utilization (month 6 and month 12) will be analysed using appropriate regression models. Intervention type, study site and utilization costs at baseline will be included as independent variables.

In a second step, cost-effectiveness analyses of the intervention will be carried out from the healthcare perspective. Therefore, the development of the different endpoints (see above) will be analysed in relation to the costs of health-care utilization and the costs of the intervention. Incremental cost effectiveness ratios and corresponding confidence intervals will be estimated.

15 Patient Involvement

Literature on needs and motivation of patients that are comparable to the patients addressed in this trial has been reviewed and results are integrated in the trial design. To involve patients directly, the patient organization *Gesundheit Aktiv* has conducted a call to all members for contacting the Coordinating Investigator to give input of the patient perspective into the trial planning. Also representative of senior citizens, geriatric nurses, therapists, Tai Chi teacher, primary care physicians were contacted. Personal semi-standard interviews were then conducted with patients, nurses, geriatric nurses, therapists and Tai Chi teachers, and primary care doctors providing care to patients. In these interviews, the trial design, inclusion criteria, recruitment procedures, random allocation, standard care, exercise classes, instructions for patients and outcome assessment were explained, and comments and recommendations were asked for. The feedback included aspects on motivation; on how to recruit older patients, patients leading a reclusive life, and people from a lower socioeconomic background; the importance of an appreciating, encouraging, calm atmosphere, giving sufficient time, avoiding the sense of being in need of help and avoiding judgmental and paternalistic communication. Also the important of providing audio-visual material to support patients practicing at home was outlined. These recommendations were included in the adaptions of the trial design and recruitment strategy, and will be met by a refresher training in appreciating, encouraging, non-judgmental communication for therapists, teachers, trainees and study nurses during investigator meetings and visit of study site.

The design, conduction, analysis and presentation of the trial will be attended and supported by patient representatives forming the patient expert group. The procedures follow the recommendations by INVOLVE:

1. National Institute for Health Research: INVOLVE. Briefing notes for researchers: public involvement in NHS, public health and social care research. February 2012. http://www.invo.org.uk/wp-content/uploads/2014/11/9938_INVOLVE_Briefing_Notes_WEB.pdf
2. National Institute for Health Research: INVOLVE. Public involvement in clinical trials: Supplement to the briefing notes for researcher. March 2012. http://www.invo.org.uk/wp-content/uploads/2015/04/INVOLVE-Clinical-trials-supplement-rev-March-2015.pdf
3. National Institute for Health Research: INVOLVE. Developing training and support for public involvement in research. http://www.invo.org.uk/wp-content/uploads/2015/06/8774-INVOLVE-Training-Support-WEB2.pdf
4. National Institute for Health Research: INVOLVE. Public Information Pack (for members of the public)
    - Booklet 1: So what is it all about?
    - Booklet 2: Getting started
    - Booklet 3: Finding out more
    - Booklet 4: Jargon buster

    http://www.invo.org.uk/posttypepublication/the-public-information-pack-pip/

Goals

The goal is to take into account the views, values, needs, abilities and limitations of the patients

throughout the course of the study. All study procedures should be feasible for the patients. All information for patients and public should be well understandable and presented.

Membership

Members of the patient expert group are invited by the project team or can contact the project team.

- They represent at least one of the following:
    - Patients that are enrolled in the trial (age ≥ 65 years, subjectively increased risk of falling).
    - Close caregiver or care provider of the patient population (e.g. geriatric nursing care).
    - Organization representing interests of these patients (e.g. senior citizens' organization).
- Memberships is free and may be terminated at any time by the member without giving reasons, or it ends with the end of the trial.
- The members should have interest in research.
- The members are partner of the study team.

Responsibilities

- The members introduce and share their subjective expertise of patient’s view, needs and values into study design and conduction.
- Confidential documents or information will be kept confidential.

Tasks, meetings

- All relevant trial documents will be send to the members, they are invited to comment and make suggestions:
    - The trial protocol and a generally understandable German summary.
    - Several written evidence-based information on fall prevention to be handed out to patients; members will give recommendations on which to choose.
    - All information and brochures handed out to patients (including website texts); audiovisual material for practicing.
    - Interim reports that describe the progress of the trial *.

- Results of the analysis, study reports, articles about the trial to be published, presentation for the public *.

  * The members can receive a German summary

- Members will be invited to important trial meetings and investigator meetings. Relevant documents will be sent to them at least a week ahead.
- They will be informed in personal or telephone/internet meetings about the trial and its progress; questions will be answered and unclear points clarified.
- They will be informed about unexpected problems of trial conduction.
- They will be consulted regarding the planned announcement and patient recruitment, particularly of patients which are economically disadvantaged or living a reclusive life.

Documentation, Reimbursement, Acknowledgement, Authorship

Meetings, personal and written communication and advices given by the members will be documented.

The members expenses will be reimbursed, following recommendation of INVOLVE. They will be supported by all members of the study team upon request at any time. They will be recognized and named in trial publications and can potentially become co-authors if fulfilling the requirement of authorship (http://www.icmje.org/recommendations/browse/roles-and-responsibilities/defining-the-role-of-authors-and-contributors.html)

16 Ethical and Legal Principles

16.1 Subject Informed Consent

Before enrolment in the clinical trial, the subject will be informed that participation is voluntary and that he/she may withdraw at any time without having to give reasons and without penalty or loss of benefits to which the subject is otherwise entitled.

The subject will be given ample time and opportunity to obtain answers to any open questions. All questions should be answered to the satisfaction of the subject and/or his/her legal representatives. In addition, the subject will be given a "Patient Informed Consent Form“, which contains all the important information in writing.

The subject's written consent must be obtained before any study-specific tests.

For this purpose, the written consent form will be personally dated and signed

by the study subject/both legal representatives and the physician conducting the informed consent discussion.

By signing the consent form, the subject agrees to voluntarily participate in the trial and declares that he/she agrees to be contacted regularly, to follow-up visits and If applicable regular group sessions.

After signing, the subject will be given one copy (or another original) of the signed and dated written consent form and any other written information to be provided to the subjects.

16.2 Ethical and Regulatory Requirements

In Germany, a favourable opinion from each competent ethic committee must be obtained before a study is started (see also § 15 *(Muster-)Berufsordnung für die in Deutschland tätigen Ärztinnen und Ärzte*) . The “Sponsor” (according to BMBF) will apply on behalf of the investigator for the positive opinion of each competent ethic committee. In Germany all investigators (including sub-investigators joining the team) subsequently have to provide their relevant investigator qualification documents to the ”Sponsor”.

16.3 Data Protection and Confidentiality

The pertinent provisions on data protection must be fully complied with.

The study subjects will be informed of the purpose and extent of the collection and use of personal data, particularly medical data.

Findings obtained in the course of the study will be stored on electronic media and treated in strict confidence. For the protection of these data, organisational measures have been taken to prevent disclosure to unauthorised third parties. For example, the subject data will be captured

in pseudonymised form (subject ID No. for the particular study, year of birth) throughout the documentation and evaluation phase.

A data protection concept for this clinical trial is available at the Clinical Trials Unit, Medical Center – University of Freiburg.

16.4 Archiving

After completion of the clinical trial, the subject identification log and all essential study documents will be retained at the study site for ten years. The investigator will be responsible for the storage.

17 Registry

The coordinating investigator assures that the key design elements of this protocol will be posted in a publicly accessible clinical trials registry such as DRKS. In addition, upon trial completion the results of this trial will be submitted for publication and/or posted in a publicly accessible database of clinical trial results.

Reporting guidelines will be taken into account (see www.equator-network.org), e.g. the CONSORT statement should be adhered to in the preparation of papers on the results of randomised studies.

Open Data

The principle of „Open Data“ is supported, following the recommendations of the *Berlin Institute of Health* (BIH) (https://www.bihealth.org/de/quest-center/mission-ansaetze/open-science). Anonymised data may be shared with cooperating scientists or scientists who have other medically or scientifically well-founded reasons (goals referring to transparency, replicability, different analyses, combinations and meta-analyses of data). Their research must aim at improving care of elderly people, and they have to ensure data protection.

18 Financing of the project

18.1 Financing

The project is funded by the Federal Ministry of Education and Research of Germany

18.2 Study Reports

After completion of the analysis by the responsible biostatistician, the coordinating investigator together with the study team will prepare the study report. The coordinating investigator will sign the final integrated medical and statistical report jointly with the biostatistician.

Except when required by law, no one will disclose a result of the study to third parties unless all parties involved have first agreed on the results of the analysis and their interpretation.

18.3 Publication of Study Protocol and Results

The coordinating investigator assures that the key design elements of this protocol will be posted in a publicly accessible clinical trials registry such as DRKS. In addition, upon study completion the results of this study will be submitted for publication.

Reporting guidelines will be taken into account (see www.equator-network.org), e.g. the CONSORT statement should be adhered to in the preparation of papers on the results of randomised studies.

Each publication of study results will be in mutual agreement between the principal investigator, the other investigators involved and the Clinical Trials Unit. All data collected in connection with the clinical study will be treated in confidence by the coordinating investigator and all others involved in the study, until publication. Interim data and final results may only be published (orally or in writing) with the agreement of the coordinating investigator and the Clinical Trials Unit. This is indispensable for a full exchange of information between the above-named parties, which will ensure that the opinions of all parties involved have been heard before publication. The agreement, which does not include any veto right or right of censorship for any of the parties involved, may not be refused without good reason.

19 References

19.1 Relevant Guidelines and Laws

Declaration of Helsinki	http://www.wma.net/en/30publications/10policies/b3/
ICH E6 (R2) - GCP Guideline	http://www.ich.org/products/guidelines/efficacy/efficacy-single/article/good-clinical-practice.html
ICH E8 – General considerations for clinical trials	http://www.ich.org/products/guidelines/efficacy/efficacy-single/article/general-considerations-for-clinical-trials.html

19.2 References

[1] Denkinger MD, Lukas A, Herbolsheimer F, Peter R, Nikolaus T. Physical activity and other health-related factors predict health care utilisation in older adults: the ActiFE Ulm study. Z Gerontol Geriatr. 2012;45:290-7.

[2] WHO. World report on Ageing And Health. World Health Organization, Geneve2015.

[3] Böhm K, Tesch-Römer C, Ziese T, (Eds). Gesundheit und Krankheit im Alter. Beiträge zur Gesundheitsberichterstattung des Bundes. Berlin: Robert Koch Institut; 2009.

[4] Angevaren M, Aufdemkampe G, Verhaar HJ, Aleman A, Vanhees L. Physical activity and enhanced fitness to improve cognitive function in older people without known cognitive impairment. Cochrane Database Syst Rev. 2008:CD005381.

[5] Fox KR, Ku PW, Hillsdon M, Davis MG, Simmonds BA, Thompson JL, et al. Objectively assessed physical activity and lower limb function and prospective associations with mortality and newly diagnosed disease in UK older adults: an OPAL four-year follow-up study. Age Ageing. 2015;44:261-8.

[6] Steptoe A, Shankar A, Demakakos P, Wardle J. Social isolation, loneliness, and all-cause mortality in older men and women. Proc Natl Acad Sci U S A. 2013;110:5797-801.

[7] Gillespie LD, Robertson MC, Gillespie WJ, Sherrington C, Gates S, Clemson LM, et al. Interventions for preventing falls in older people living in the community. Cochrane Database Syst Rev. 2012:CD007146.

[8] Kendrick D, Kumar A, Carpenter H, Zijlstra GA, Skelton DA, Cook JR, et al. Exercise for reducing fear of falling in older people living in the community. Cochrane Database Syst Rev. 2014:CD009848.

[9] Howe TE, Rochester L, Neil F, Skelton DA, Ballinger C. Exercise for improving balance in older people. Cochrane Database Syst Rev. 2011:CD004963.

[10] Wayne PM, Fuerst ML. The Harvard Medical School Guide to Tai Chi: 12 Weeks to a Healthy Body, Strong Heart, and Sharp Mind Boston, London: Shambhala; 2013.

[11] Harris PE, Cooper KL, Relton C, Thomas KJ. Prevalence of complementary and alternative medicine (CAM) use by the general population: a systematic review and update. Int J Clin Pract. 2012;66:924-39.

[12] Kienle GS, Kiene H, Albonico HU. Anthroposophic Medicine: Effectiveness, Utility, Costs, Safety. Stuttgart, New York: Schattauer Verlag; 2006.

[13] Lotzke D, Heusser P, Bussing A. A systematic literature review on the effectiveness of eurythmy therapy. J Integr Med. 2015;13:217-30.

[14] Marengoni A, Angleman S, Melis R, Mangialasche F, Karp A, Garmen A, et al. Aging with multimorbidity: a systematic review of the literature. Ageing Res Rev. 2011;10:430-9.

[15] Lawlor DA, Patel R, Ebrahim S. Association between falls in elderly women and chronic diseases and drug use: cross sectional study. BMJ. 2003;327:712-7.

[16] Rapp K, Freiberger E, Todd C, Klenk J, Becker C, Denkinger M, et al. Fall incidence in Germany: results of two population-based studies, and comparison of retrospective and prospective falls data collection methods. BMC Geriatr. 2014;14:105.

[17] Li F, Harmer P. Economic Evaluation of a Tai Ji Quan Intervention to Reduce Falls in People With Parkinson Disease, Oregon, 2008-2011. Prev Chronic Dis. 2015;12:E120.

[18] Scheffer AC, Schuurmans MJ, van Dijk N, van der Hooft T, de Rooij SE. Fear of falling: measurement strategy, prevalence, risk factors and consequences among older persons. Age Ageing. 2008;37:19-24.

[19] Kiel DP. Falls in older persons: Risk factors and patient evaluation. In: Schmader KE, editor. UpToDate Waltham, MA: UpToDate Inc http://www.uptodate.com2018.

[20] Haagsma JA, Graetz N, Bolliger I, Naghavi M, Higashi H, Mullany EC, et al. The global burden of injury: incidence, mortality, disability-adjusted life years and time trends from the Global Burden of Disease study 2013. Inj Prev. 2016;22:3-18.

[21] Visschedijk J, Achterberg W, Van Balen R, Hertogh C. Fear of falling after hip fracture: a systematic review of measurement instruments, prevalence, interventions, and related factors. J Am Geriatr Soc. 2010;58:1739-48.

[22] Kiel DP, Schmader KE, Libman H. Falls: Prevention in community-dwelling older persons. . In: Post TW, editor. UpToDate®. Waltham, MA2017.

[23] Blumenberg P, Büscher A, Krebs M, Moers M, Möller A, Schiemann D, et al. Expertenstandard Sturzprophylaxe in der Pflege. In: (DNQP) DNfrQtidP, editor. Osnabrück: Hochschule Osnabrück; 2013.

[24] Becker C, Brenner N, Blessing-Kapelke U. Trittsicher durchs Leben - Gleichgewicht & Kraft. Einführung in die Sturzprävention. http://www.trittsicher.org/files/trittsicher_bzga_sturzpraevention_2015-11-23.pdf.

[25] Krug S, Jordan S, Mensink GB, Muters S, Finger J, Lampert T. Körperliche Aktivität: Ergebnisse der Studie zur Gesundheit Erwachsener in Deutschland (DEGS1). Bundesgesundheitsblatt Gesundheitsforschung Gesundheitsschutz. 2013;56:765-71.

[26] Gardner B, Thune-Boyle I, Iliffe S, Fox KR, Jefferis BJ, Hamer M, et al. 'On Your Feet to Earn Your Seat', a habit-based intervention to reduce sedentary behaviour in older adults: study protocol for a randomized controlled trial. Trials. 2014;15:368.

[27] Hill KD, Day L, Haines TP. What factors influence community-dwelling older people's intent to undertake multifactorial fall prevention programs? Clin Interv Aging. 2014;9:2045-53.

[28] Schreier MM, Bauer U, Osterbrink J, Niebauer J, Iglseder B, Reiss J. Fitness training for the old and frail. Effectiveness and impact on daily life coping and self-care abilities. Z Gerontol Geriatr. 2016;49:107-14.

[29] Stevens JA, Voukelatos A, Ehrenreich H. Preventing falls with Tai Ji Quan: A public health perspective. J Sport Health Sci. 2014;3:21-6.

[30] Hamre HJ, Witt CM, Glockmann A, Ziegler R, Willich SN, Kiene H. Eurythmy therapy in chronic disease: a four-year prospective cohort study. BMC Public Health. 2007;7:61.

[31] Kanitz JL, Pretzer K, Calaminus G, Wiener A, Langler A, Henze G, et al. Eurythmy therapy in the aftercare of pediatric posterior fossa tumour survivors--a pilot study. Complement Ther Med. 2013;21 Suppl 1:S3-9.

[32] Ritchie J, Wilkinson J, Gantley M, Feder G, Carter Y, Formby J. A model of integrated primary care: anthroposophic medicine. London: National Centre for Social ResearchDepartment of General Practice and Primary Care, StBartholomew's and the Royal London School of Medicine and Dentistry, Queen Mary, University of London. 2001;158.

[33] Clarke TC, Black LI, Stussman BJ, Barnes PM, Nahin RL. Trends in the use of complementary health approaches among adults: United States, 2002-2012. Natl Health Stat Report. 2015:1-16.

[34] Linde K, Alscher A, Friedrichs C, Joos S, Schneider A. Die Verwendung von Naturheilverfahren, komplementaren und alternativen Therapien in Deutschland - eine systematische Ubersicht bundesweiter Erhebungen. Forsch Komplementmed. 2014;21:111-8.

[35] Eardley S, Bishop FL, Prescott P, Cardini F, Brinkhaus B, Santos-Rey K, et al. A systematic literature review of complementary and alternative medicine prevalence in EU. Forsch Komplementmed. 2012;19 Suppl 2:18-28.

[36] Salkeld G, Cameron ID, Cumming RG, Easter S, Seymour J, Kurrle SE, et al. Quality of life related to fear of falling and hip fracture in older women: a time trade off study. BMJ. 2000;320:341-6.

[37] Tinetti ME, Williams CS. The effect of falls and fall injuries on functioning in community-dwelling older persons. J Gerontol A Biol Sci Med Sci. 1998;53:M112-9.

[38] Jorstad EC, Hauer K, Becker C, Lamb SE, ProFa NEG. Measuring the psychological outcomes of falling: a systematic review. J Am Geriatr Soc. 2005;53:501-10.

[39] Heinrich S, Rapp K, Rissmann U, Becker C, Konig HH. Cost of falls in old age: a systematic review. Osteoporos Int. 2010;21:891-902.

[40] Sherrington C, Fairhall NJ, Wallbank GK, Tiedemann A, Michaleff ZA, Howard K, et al. Exercise for preventing falls in older people living in the community. Cochrane Database Syst Rev. 2019;1:CD012424.

[41] Sherrington C, Tiedemann A, Fairhall N, Close JC, Lord SR. Exercise to prevent falls in older adults: an updated meta-analysis and best practice recommendations. N S W Public Health Bull. 2011;22:78-83.

[42] Unfallversicherungsanstalt A. Forschungsbericht BAUfit - Beratungs- und Trainingsprogramme für Baufirmen. AUVA-Projekt 1999-2000. Endbericht. Nummer 38. 2004.

[43] Kroz M, Reif M, Glinz A, Berger B, Nikolaou A, Zerm R, et al. Impact of a combined multimodal-aerobic and multimodal intervention compared to standard aerobic treatment in breast cancer survivors with chronic cancer-related fatigue - results of a three-armed pragmatic trial in a comprehensive cohort design. BMC Cancer. 2017;17:166.

[44] Seifert G, Kanitz JL, Pretzer K, Henze G, Witt K, Reulecke S, et al. Improvement of circadian rhythm of heart rate variability by eurythmy therapy training. EvidBased Complement AlternatMed. 2013;2013:564340.

[45] Kanitz JL, Pretzer K, Reif M, Voss A, Brand R, Warschburger P, et al. The impact of eurythmy therapy on stress coping strategies and health-related quality of life in healthy, moderately stressed adults. Complement Ther Med. 2011;19:247-55.

[46] Guirguis-Blake JM, Michael YL, Perdue LA, Coppola EL, Beil TL. Interventions to Prevent Falls in Older Adults: Updated Evidence Report and Systematic Review for the US Preventive Services Task Force. JAMA. 2018;319:1705-16.

[47] Wayne PM, Berkowitz DL, Litrownik DE, Buring JE, Yeh GY. What do we really know about the safety of tai chi?: A systematic review of adverse event reports in randomized trials. Arch Phys Med Rehabil. 2014;95:2470-83.

[48] Li F, Harmer P, Fitzgerald K, Eckstrom E, Akers L, Chou LS, et al. Effectiveness of a Therapeutic Tai Ji Quan Intervention vs a Multimodal Exercise Intervention to Prevent Falls Among Older Adults at High Risk of Falling: A Randomized Clinical Trial. JAMA Intern Med. 2018;178:1301-10.

[49] Kempen GI, Yardley L, van Haastregt JC, Zijlstra GA, Beyer N, Hauer K, et al. The Short FES-I: a shortened version of the falls efficacy scale-international to assess fear of falling. Age Ageing. 2008;37:45-50.

[50] Berg KO, Wood-Dauphinee SL, Williams JI, Maki B. Measuring balance in the elderly: validation of an instrument. Can J Public Health. 1992;83 Suppl 2:S7-11.

[51] Nasreddine ZS, Phillips NA, Bedirian V, Charbonneau S, Whitehead V, Collin I, et al. The Montreal Cognitive Assessment, MoCA: a brief screening tool for mild cognitive impairment. J Am Geriatr Soc. 2005;53:695-9.

[52] Sheikh JI, Yesavage JA. Geriatric Depression Scale (GDS): recent evidence and development of a shorter version. Clinical Gerontologist. 1986;5:165-73.

[53] Ware J, Jr., Kosinski M, Keller SD. A 12-Item Short-Form Health Survey: construction of scales and preliminary tests of reliability and validity. Med Care. 1996;34:220-33.

[54] Lawton MP, Brody EM. Assessment of older people: self-maintaining and instrumental activities of daily living. Gerontologist. 1969;9:179-86.

[55] Bussing A, Edelhauser F, Weisskircher A, Fouladbakhsh JM, Heusser P. Inner Correspondence and Peacefulness with Practices among Participants in Eurythmy Therapy and Yoga: A Validation Study. Evid Based Complement Alternat Med. 2011;2011.

[56] Seidl H, Bowles D, Bock JO, Brettschneider C, Greiner W, Konig HH, et al. FIMA – Fragebogen zur Erhebung von Gesundheitsleistungen im Alter: Entwicklung und Pilotstudie. Gesundheitswesen. 2015;77:46-52.

[57] Lamb SE, Jorstad-Stein EC, Hauer K, Becker C, Prevention of Falls Network E, Outcomes Consensus G. Development of a common outcome data set for fall injury prevention trials: the Prevention of Falls Network Europe consensus. J Am Geriatr Soc. 2005;53:1618-22.

[58] Park SH, Lee YS. The Diagnostic Accuracy of the Berg Balance Scale in Predicting Falls. West J Nurs Res. 2016:193945916670894.

[59] Shumway-Cook A, Baldwin M, Polissar NL, Gruber W. Predicting the probability for falls in community-dwelling older adults. Phys Ther. 1997;77:812-9.

[60] Bergert FW, BRaun M, Feßler J, Hüttner U, Kluthe B, Popert U, et al. Hausärztliche Leitlinie. Geriatrisches Assessment in der Hausarztpraxis. https://www.awmf.org/uploads/tx_szleitlinien/053-015l_S1_Geriatrisches_Assessment_in_der_Hausarztpraxis_2018-05.pdf: Deutsche Gesellschaft für Allgemeinmedizin und Familienmedizin (DEGAM), Berlin; Arbeitsgemeinschaft der Wissenschaftlichen Medizinischen Fachgesellschaften, AWMF. ; 2017.

[61] Scherer M, Wagner HO, Lühmann D, Muche-Borowski C, Schäfer I, Dubben HH, et al. Multimorbidität. S3-Leitlinie. https://www.awmf.org/uploads/tx_szleitlinien/053-047l_S3_Multimorbiditaet_2018-01.pdf: Deutsche Gesellschaft für Allgemeinmedizin und Familienmedizin (DEGAM), Berlin; Arbeitsgemeinschaft der Wissenschaftlichen Medizinischen Fachgesellschaften, AWMF. ; 2017.

[62] Bergert FW, Braun M, Ehrenthal K, Feßler J, Gross J, Hüttner U, et al. Hausärztliche Leitlinie. Multimedikation. https://www.awmf.org/uploads/tx_szleitlinien/053-043l_S2e_Multimedikation_2014-05.pdf: Deutsche Gesellschaft für Allgemeinmedizin und Familienmedizin (DEGAM), Berlin; Arbeitsgemeinschaft der Wissenschaftlichen Medizinischen Fachgesellschaften, AWMF. ; 2014.

[63] Li F, Harmer P, Fisher KJ, McAuley E, Chaumeton N, Eckstrom E, et al. Tai Chi and fall reductions in older adults: a randomized controlled trial. J Gerontol A Biol Sci Med Sci. 2005;60:187-94.
